# Solvent-Dependent Structural Dynamics in the Ultrafast Photodissociation Reaction of Triiodide Observed with Time-Resolved X-ray Solution Scattering

**DOI:** 10.1021/jacs.3c00484

**Published:** 2023-05-10

**Authors:** Amke Nimmrich, Matthijs R. Panman, Oskar Berntsson, Elisa Biasin, Stephan Niebling, Jonas Petersson, Maria Hoernke, Alexander Björling, Emil Gustavsson, Tim B. van Driel, Asmus O. Dohn, Mads Laursen, Diana B. Zederkof, Kensuke Tono, Tetsuo Katayama, Shigeki Owada, Martin M. Nielsen, Jan Davidsson, Jens Uhlig, Jochen S. Hub, Kristoffer Haldrup, Sebastian Westenhoff

**Affiliations:** † Department of Chemistry and Molecular Biology, University of Gothenburg, Box 462, 40530 Gothenburg, Sweden; ‡ Department of Physics, Technical University of Denmark, 2800 Lyngby, Denmark; ¶ Department of Chemistry, Ångström Laboratory, Uppsala University, Box 523, 75120 Uppsala, Sweden; § Faculty of Physical Sciences, University of Iceland, VR-III, 107 Reykjavík, Iceland; ∥ Japan Synchrotron Radiation Research Institute, 1-1-1 Kouto, Sayo-cho, Sayo-gun, Hyogo 679-5198, Japan; ⊥ RIKEN SPring-8 Center, 1-1-1 Kouto, Sayo-cho, Sayo-gun, Hyogo 679-5148, Japan; # Department of Chemical Physics, Lund University, Box 124, 22100 Lund, Sweden; ◐ Georg-August-Universität Göttingen, Institute for Microbiology and Genetics, Justus-von-Liebig-Weg 11, 37077 Göttingen, Germany

## Abstract

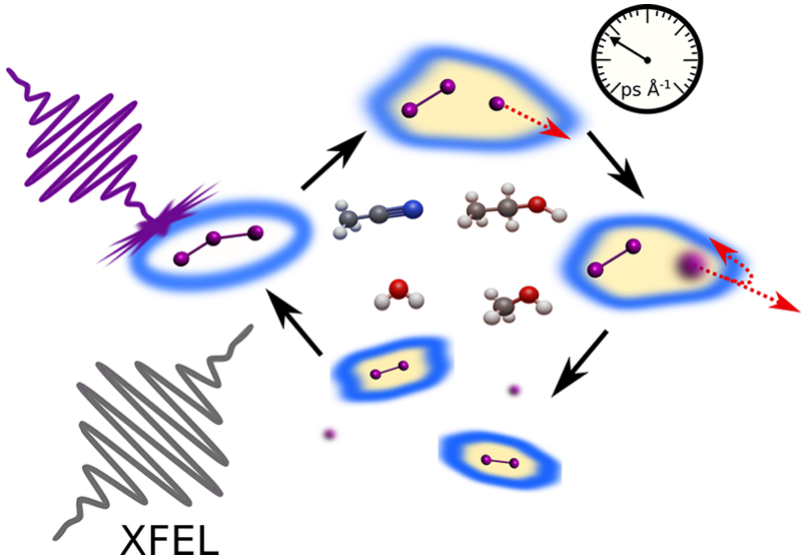

Resolving the structural dynamics of bond breaking, bond formation, and solvation is required for a deeper understanding of solution-phase chemical reactions. In this work, we investigate the photodissociation of triiodide in four solvents using femtosecond time-resolved X-ray solution scattering following 400 nm photoexcitation. Structural analysis of the scattering data resolves the solvent-dependent structural evolution during the bond cleavage, internal rearrangements, solvent-cage escape, and bond reformation in real time. The nature and structure of the reaction intermediates during the recombination are determined, elucidating the full mechanism of photodissociation and recombination on ultrafast time scales. We resolve the structure of the precursor state for recombination as a geminate pair. Further, we determine the size of the solvent cages from the refined structures of the radical pair. The observed structural dynamics present a comprehensive picture of the solvent influence on structure and dynamics of dissociation reactions.

## Introduction

Ultrafast photoinduced chemical reactions in solution and condensed-phase environments are the underlying phenomena for many processes, e.g., for photosynthesis and vision in biological systems as well as for photocatalysis and for dye-sensitized solar cells. Despite the importance of such reactions, the influence of the solvent environment on structural dynamics in solution-phase reaction is not yet fully understood.^1−3^ Solvent–solute interactions are known to affect the potential energy landscapes, a phenomenon which can be experimentally observed e.g., by shifts in absorption spectra and changes in lifetimes of different electronic states as a function of solvent type.^4−6^ The solvent is therefore also expected to influence the earliest dynamics in solution-phase reactions, fundamentally impacting their course. To understand the solvent effect on the subpicosecond reaction events, the direct observation of reactions on these time scales is necessary. This is made possible by investigating photoinitiated reactions with time-resolved scattering methods.

Over the last few decades, transient optical spectroscopy on subpicosecond time scales enabled the observation of photoinduced reactions in real time by probing the population of different electronic states at different time points throughout a reaction.^7^ Optical spectroscopy is however not directly sensitive to structure. Using X-ray scattering such structural information can be obtained since the measured scattering patterns arise directly as a function of the molecular structure(s). The first time-resolved X-ray scattering experiments on ultrafast time scales were performed at synchrotrons, giving structural insight on a subnanosecond time scale.^8−10^ X-ray free electron lasers (XFELs)^11^ combined the advantages of high-brilliance X-ray sources with subpicosecond probe pulse lengths similar to optical spectroscopy, opening the possibility for ultrafast X-ray spectroscopy^12^ and ultrafast Time-resolved X-ray solution scattering (TR-XSS)^13,14^ as recently reviewed.^15−17^ The development of TR-XSS at XFELs now allows for subpicosecond and sub-Ångström determination of the intramolecular structural dynamics of a solute^15,16,18^ as well as detailed studies of the solvent rearrangement and dynamics surrounding photoexcited reactants.^19−21^

A class of chemical reactions that has been widely investigated as models for fundamental reaction events are photoinitiated dissociation reactions, where bond cleavage in a molecule is induced by irradiation with light in the UV to visible range. Photodissociation reactions, such as the dissociation of diiodomethane,^22^ geminal trihalides,^23,24^ or ICN,^25,26^ have been widely used to investigate fundamental reaction events, including bond cleavage,^27,28^ the possibility of fragments escaping the solvent cage,^27,28^ or roaming pathways leading to isomer formation,^29,30^ followed by geminate or nongeminate recombination.^27,28,31^ In some cases, geminate recombination was further subdivided into primary and secondary geminate recombination. The former describes the recombination of fragments directly inside the first layer of solvent molecules within a few picoseconds. In secondary geminate recombination, the fragments are separated by a few layers of solvent molecules, also referred to as “contact pair”, leading to recombination on tens to hundreds of picoseconds.^32−35^ A model system which has been utilized to observe ultrafast bond cleavage and recombination has been the photodissociation of triiodide, I_3_^–^ + *h*ν → I_2_^–^ + I.^36−39^ Due to its seemingly simple dissociation reaction involving only three solute atoms, it has been extensively investigated using a manifold of techniques, including absorption,^36,39,40^ Raman,^41^ and photoelectron spectroscopy^42^ as well as TR-XSS^43^ and electron diffraction.^44^

The photodissociation of I_3_^–^ (I_3_^–^ → I_2_^–^ + I) takes place upon photoexcitation into one of the two broad absorption bands centered around 290 and 360 nm (Figure S1). Previous studies suggest that, at 400 nm, as used in the present study, only the two-body dissociation reaction takes place.^45^ In gas phase as well as in solution phase when exciting in the 290 nm band, additional contributions from three-body dissociation are also observed.^39,46−48^ From optical spectroscopy, photoinduced bond breaking in I_3_^–^ is known to take place within 400 fs based on the appearance of characteristic spectral signatures of the I_2_^–^ fragment on this time scale.^36,38,49^ Subsequently, the ground state is repopulated on three distinct, solvent-dependent time scales (τ_1_ = 1 to 5.4 ps, τ_2_ = 12 to 91 ps, τ_3_ > 350 ps), observed as decay of the ground state bleach.^38,50,51^ In these studies, the short lifetime τ_1_ was assigned to direct recombination of a so-called geminate pair (GP) with the photofragments remaining trapped inside the solvent cage of the parent I_3_, while the longer lifetime τ_3_ was assigned to nongeminate (NG) recombination of solvent-separated fragments. From the fraction of fragments recombining on the longer time scale, described by τ_3_, the probability of cage escape could be estimated and was found to be strongly solvent dependent and dependent on the solvent molecule size and intermolecular forces such as van der Waal’s interactions and hydrogen bonding.^37,40,50^ The intermediate lifetime, τ_2_, was observed in several different studies in a range of solvents,^39,50,52^ in some cases showing a slightly shifted absorption spectrum compared to free I_2_^–^,^38,50^ but neither the electronic character nor the structure of the reaction intermediate could be uniquely assigned to this lifetime. As such, τ_2_ was often simply assigned to a “state X”. Studies in ionic liquids^37^ and temperature-dependent experiments in ethanol^51^ showed that the presence of state X did not depend on the fraction of excited state species decaying with the τ_3_ lifetime, indicating state X is not a solvent-separated species.

Of direct relevance to the present structural study of solvent effects, not only the dynamics of the photodissociation but also the structure of the ground state of I_3_^–^ itself has been found to be solvent dependent. Spectroscopic studies showed features from asymmetric vibrations of I_3_^–^ in protic solvents while these were not visible in apolar solvents.^41,53−55^ Later structural studies using X-ray techniques and molecular dynamics simulations demonstrated that the bending angle and bond lengths of I_3_^–^ depend on the surrounding solvent.^56−58^ However, these studies did not reach agreement in terms of the molecular structure of ground state triiodide, showing varying bond distances and bending angles. The exact ground state structures are hence still a topic of discussion.^42,43^

Most recently, two studies of the I_3_^–^ → I_2_^–^ + I reaction in solution utilizing ultrafast scattering techniques with subpicosecond time resolution have emerged. In a pioneering study,^44^ Ledbetter et al. utilized liquid-phase ultrafast MeV electron diffraction with ∼180 fs time resolution (fwhm) to estimate the dissociation speed following the photoexcitation event and found this to be around *v*_diss._ = 5.8(3) Å/ps in water. In the same study, the lifetime of the geminate pair was estimated to be 0.6(3) ps and the cage-escape ratio was 0.26(10). Adding to these results, a 2022 study by Heo et al. utilized TR-XSS at the PAL-XFEL X-ray free electron laser to obtain experimental data with 0.3 ps time resolution (fwhm) on the I_3_^–^ → I_2_^–^ + I reaction in methanol.^43^ The authors refined an asymmetric (*R*_I–I_ = 3.09/2.96 Å) and nonlinear (α = 152°) ground state structure of I_3_^–^ and demonstrate a refinement of the charge distribution on the molecule as a −1 excess charge strongly localized on the long-bond I atom. Analysis of the reaction kinetics and structural dynamics indicated that it was the longer bond which preferentially was broken and that the initial speed of fragment dissociation was 5.6 Å ps^–1^ with a cage-escape ratio of 0.42(11). Geminate recombination in methanol was found to take place on two time scales, τ_GP–rec._ = 3.1(6) and 49(22) picoseconds.

In this work, we employ TR-XSS to directly investigate the solvent-dependent structural dynamics of the photodissociation and following recombination of triiodide in four different solvents. The contributions to the experimental signals arising from changes in the solvent cage structures were explicitly included in our model by establishing a large library of cage structures around different solute structures during the I_3_^–^ → I_2_^–^ + I reaction. For all solvents, the speed of dissociation is determined along with the cage escape ratios and the intramolecular dynamics of the I_2_^–^ fragment via structural refinement.

## Methods

### Experiment

**Figure 1:**
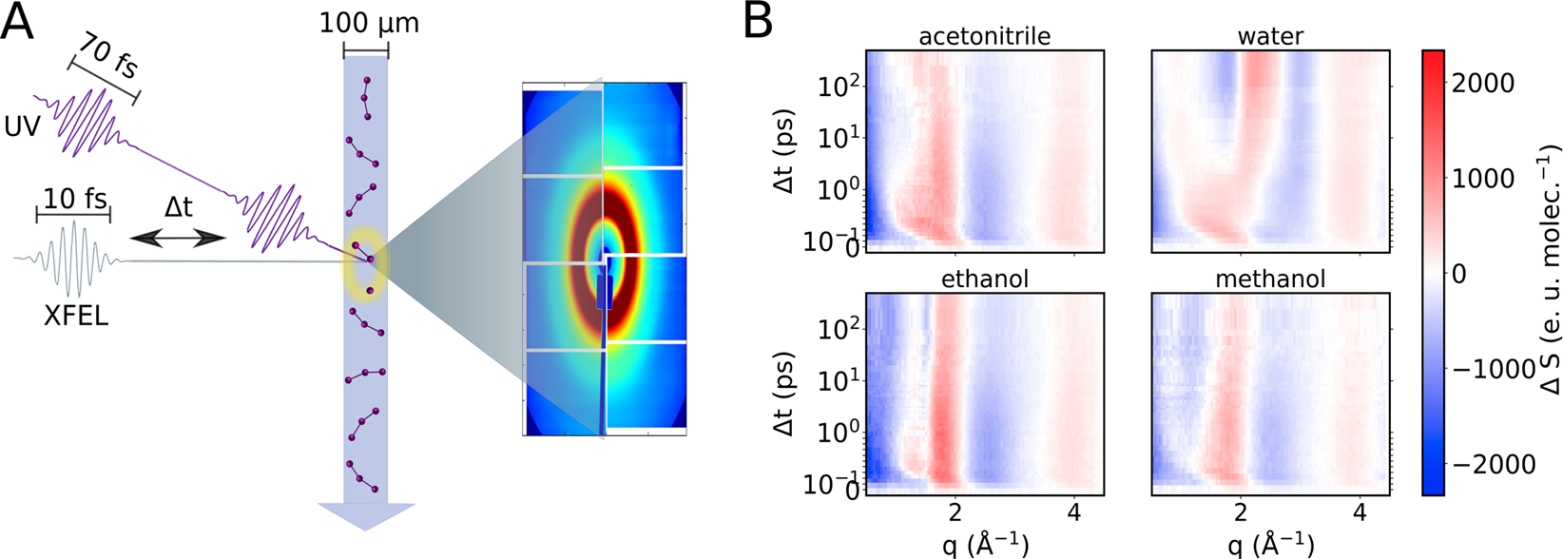
(A) Schematic representation of the experimental setup. The XFEL pulse and laser pump pulse (400 nm, 70 fs fwhm) are overlapped at the sample position. Scattering patterns are recorded with the octal MPCCD detector. (B) The time-dependent difference scattering signals for the four solvents studied (top left to bottom right: acetonitrile, water, ethanol, and methanol) are presented. The data covers time delays from −0.1 to 500 ps and a *q*-range of 0.5 to 4.5 Å^–1^.

The experiments reported on here were performed as laser pump/X-ray probe implemented at the SPring-8 Ångstrom Compact free electron LAser (SACLA) as schematically illustrated in Figure 1A. The XFEL probe beam was defined by slits to be 300 μm fwhm at the sample position, had an energy of 11.98 ± 0.028 keV and a temporal length of ∼10 fs fwhm, and was delivered with 30 Hz repetition rate. The X-ray beam was overlapped with the pump laser beam (400 nm, ∼450 μm fwhm spot size, 70 fs fwhm, 450 μJ pulse energy, giving a fluence of ∼2.3 mJ/mm^2^) at the sample position, about 300 μm below a rectangular sapphire nozzle providing a liquid jet with a thickness of 100 μm. Experiments were performed in four different solvents (acetonitrile, water, ethanol, methanol) and with an I_3_^–^ concentration around 9.2 mmol L^–1^; see the Supporting Information (SI).

The scattering patterns were detected using the MPCCD Octal Sensor Detector.^59^ The raw data were corrected for X-ray polarization, solid-angle coverage, and background contributions to yield 2D *S*(*q*, ϕ) scattering data for each shot, where *q* is the scattering vector  and ϕ is the azimuthal angle on the detector.^19^ These 2D patterns were subsequently binned as a function of time delay *t* between the laser and X-ray with pump–probe jitter and drift corrected with information from the Arrival Time Monitor diagnostic installed at the beamline.^60^ Since the photoinduced changes in *S*(*q*, ϕ) are less than a few percent of the total signal, it is convenient to extract the difference scattering signal Δ*S* for our analysis to isolate the photoinduced changes in scattering. To determine these difference scattering signals, every seventh X-ray shot was without laser excitation. The nearest ten “dark” scattering patterns to a “light” measurement were then averaged and subtracted (Δ*S*(*q*) = *S*_on_ – *S*_off_).

Due to the linear polarization of the optical laser pulses, anisotropic contributions to the scattering patterns arise from the relative orientation of the excitation polarization and the transition dipole moment. To separate isotropic and anisotropic contributions to the scattering (Δ*S*_0_ and Δ*S*_2_, respectively), we follow the procedure previously presented^61^ with radial integration performed in 15 azimuthal sections/slices. In the final step of the data reduction, the difference signals were put on an absolute scale (e.u. molec.^–1^) determined by scaling azimuthally integrated scattering patterns *S*(*q*) to the simulated signal from one liquid unit cell (the smallest stoichiometrically representative unit; see Figure S4). Figure 1B shows the time-resolved difference scattering data for all solvents covering a *q*-range of 0.5 to 4.6 Å^–1^. Data is shown on a linear time scale for the first 1 ps, after which a logarithmic scale is used.

To determine possible contributions from multiphoton absorption processes to the difference scattering signal, measurements at different excitation energies ranging from 50 to 600 μJ were performed, as detailed in Figures S5 and S6. We chose the laser power of 450 μJ in order to retain good signal-to-noise in the limited experimental time frame while minimizing detrimental adverse effects to the scattering signal or dynamics. The effective time resolution of the experiment is expected to be limited by the velocity mismatch of the pump and probe pulse in the sample and was estimated by fitting the time-resolved increase of the integrated difference signal with an error function, with this fit yielding a time resolution of about 175 fs (fwhm, Figure S3).

### Analysis of Difference XSS Signals

For analysis of the acquired difference XSS signals, we consider both the isotropic and anisotropic difference scattering. For modeling the isotropic difference scattering, we include three contributions to the total difference signals arising from three conceptually different sets of dynamics: intrasolute (Δ*S*_solute_), solvent cage (Δ*S*_cage_), and bulk solvent heating (Δ*S*_ΔT_). The contributions are calculated and included from sets of putative molecular configurations following photoexcitation, corresponding solvent-cage structures from MD simulations, and reference measurements of the difference signals arising from (isochoric) bulk solvent heating, respectively.^62^ Repressing the dependence on *t* and *q* for clarity of presentation, this model can be summarized as
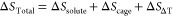
1Denoting each of the three terms here as Δ*S*_*i*_, we note that each is calculated as a difference between ground state (GS) and excited state (ES) Δ*S*_*i*_(*t*) = *S*_*i*,ES_ – *S*_*i*,GS_ and that Δ*S*_cage_ can be further subdivided into individual contributions (Δ*S*_cross_ and Δ*S*_DV_) as detailed in the following sections.

**Figure 2:**
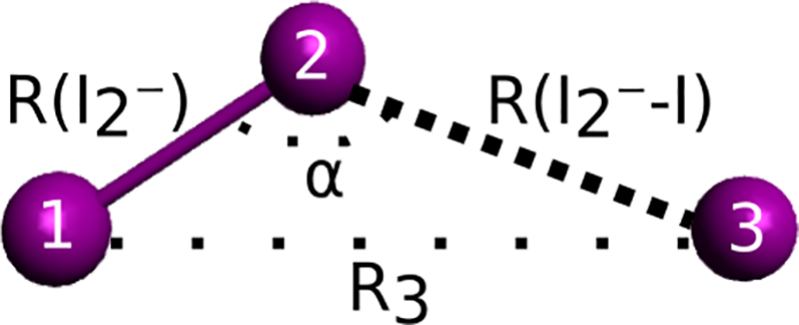
Sketch of the triiodide molecule with all structural parameters used to describe the structure. In the structural refinement, , , and α were optimized.

To calculate the difference signals from the intrasolute structural dynamics, Δ*S*_solute_, a description of the ground state (GS) structure is also needed. As described in the Introduction, the structure of I_3_^–^ in solution has been a topic of discussion over the past decade.^43,56,57,63^ Here, we use GS structures obtained from MD simulations published by Jena et al. (Table 1)^57^ with the −1 charge distributed as −0.25*e* at each of the terminal I atoms and −0.5*e* on the central atom.^49^ A comparison of different GS structures for modeling the difference scattering at 500 ps is added in Figure S10, illustrating the sensitivity to changes in the GS structure. Inclusion of the optimization of the GS structure in the refinement was however not possible here due to the correlation with other refinement parameters. To describe the structure(s) after photoexcitation, the relative arrangement of the three iodine atoms was defined by the distance  between iodine atoms **1** and **2**, forming the I_2_^–^ fragment after dissociation, the distance  between iodine atoms **2** and **3**, and the I–I–I angle α (Figure 2). In the following, we represent these structural parameters as the vector . In order to model the difference scattering, Δ*S*(*q*, **R**), the calculated scattering from a ground state structure with **R**_**GS**_ is subtracted from the scattering curves calculated for a laser induced species, which is described by the structural parameters **R**.
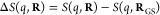
2

**Table 1:** Ground State Structures of Triiodide in Different Solvents Reported by Jena et al.^57^ and Used Here for Calculating Scattering of the GS Species

	*R*(I_2_^–^) (Å)	*R*(I_2_^–^ – I) (Å)	α (deg.)
acetonitrile	2.95	3.06	172
water	2.92	3.14	170
ethanol	2.94	3.07	171
methanol	2.94	3.09	172

To account for conformational heterogeneity in the ensemble of molecules probed by the X-ray pulses,^43,64,65^ we introduce a phenomenological Debye–Waller-like modification to the atomic form factors, , where we have followed the approach of Als-Nielsen and McMorrow (eq 5.24 in ref (66)). σ_*i*_ is the root-mean-square deviation (rmsd) of the position of atom *i* used to model the structural heterogeneity. The modified atomic form factors are then used to calculate the solute scattering via:
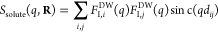
3with the atomic form factor of iodine *F*_I_ and the interatomic distance *d*_*i*,*j*_ between atom *i* and *j*. This heuristic approach to account for the heterogeneity is similar to the one taken by Heo et al.^43^ although we note that the DW-like factor in the latter work is applied to the calculation of the molecular form factor and here, to the atomic form factors. A comparison of the refinement results when applying the DW-like factor to the atomic and molecular form factor is presented in Figure S23. We assumed a constant rmsd of the atomic position estimated from MD simulations to σ_1_ = 0.5 Å, σ_2_ = 0.5 Å, and σ_3_ = 0.7 Å for the three atoms, respectively, as estimated from dynamic MD simulations (see the SI). In order to model anisotropic scattering contributions, *S*_2_(*q*), for a given solute structure, we only included solute contributions to the scattering signal following a previously published approach:^61,67^
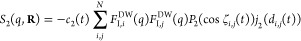
4with the amplitude *c*_2_, the second order Legendre polynomial *P*_2_, Bessel function *j*_2_, the interatomic distance *d*_*i*,*j*_, and the angle ζ_*i*,*j*_ between the vector connecting the two atoms and the transition dipole moment. Following the approach for the calculation of the isotropic scattering, we included the structural uncertainty using the modified atomic form factors *F*_I,*i*_^DW^(*q*) for the anisotropy.

Turning next to Δ*S*_cage_ arising as a consequence of primarily changes in the solvent cage structure immediately around the photoexcited solute(s), this was calculated from MD simulations. This term can be further divided into contributions from a solvent–solute cross term and a displaced volume term, both using the structure of the solvent cage from MD simulations (Δ*S*_cage_ = Δ*S*_cross_ + Δ*S*_DV_, SI). To enable computationally efficient fitting, a library of radial distribution functions (RDFs) was calculated from MD simulations as follows. We performed individual MD simulations for distances *R*(I_2_) between 2.6 and 5 Å in steps of 0.2 Å,  between 2.6 and 13.8 Å in steps of 0.2 Å, and angles α between 0 and π in steps of 0.131 (17,336 structural representations for each solvent). In each of these 4*17,336 simulations, the positions of the iodine atoms were frozen, such that the preselected arrangement **R** was maintained throughout the 1 ns long simulation trajectory. From the simulations, the RDFs between the iodine (I) and the respective solvent atom of types *v* (e.g., *v* = OH for water) and *g*_Iv_(*r*; **R**) were computed. This library of RDFs enabled the efficient calculation of the scattering arising from the solvent–solute geometry as described by Dohn et al.^68^ Changes in the solvent structure are included through a displaced volume contribution.^22^ Calculation of these two terms is described in detail in the SI along with further details on the MD simulations. Contributions to the cage term(s) from molecular structures that were not part of the MD simulation grid were calculated using trilinear interpolation between the closest library structures.^69,70^

The third term in eq 1, Δ*S*_heat_, arises as a consequence of structural changes in the bulk solvent due to isochoric heating as energy is released after photoexcitation. This contribution to the difference scattering was determined in a separate experiment for each of the four different solvents by measuring the difference signal at a time delay of 100 ps following photoexcitation of a short-lived azo-dye in the respective solvent.^62^ Bulk solvent contributions to the anisotropic scattering (optical Kerr effect) were not included in our model as their amplitude is much smaller than the observed solute contributions.^71−73^

#### Time-Dependent Structural Analysis

To determine the structural evolution following photoexcitation, we optimized the structural parameters introduced above via regularized fitting of the modeled difference signal to the experimental difference signal for both the isotropic and anisotropic difference scattering. The refinement was performed for each individual time point in each of the four data sets. In order to account for the partitioning of the fragments into geminate pair (GP, trapped inside the first solvent shell) and solvent separated pair (nongeminate pair, NG), the contributions from these two species were considered separately in the refinement. The structure of the GP species was optimized by refining the three structural parameters, **R**_**GP**_, used to calculate the difference scattering (Δ*S*_GP_). To include the difference scattering arising from the NG species (Δ*S*_NG_), the same interatomic distances for the I_2_^–^ fragment  as for the GP was assumed for each time step, but with the interfragment distance  set to 100 Å, and α = π. As such, there is no structural optimization for the NG species; only a population fraction is determined.

Assuming that each solute geometry is associated with one average cage structure, the cage term Δ*S*_cage_ can be included in the solute terms Δ*S*_0,GP_(*q*, **R**_**GP**_(*t*)) and Δ*S*_0,NG_(*q*) and the expression for total modeled scattering (1) becomes
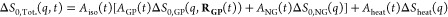
5with the amplitudes *A*_iso_, *A*_GP_, *A*_NG_ = 1 – *A*_GP_, and *A*_heat_ representing, respectively, the time-dependent excitation fraction, the fractional amount of geminate pairs, and the temperature increase of the bulk solvent. Similarly, the anisotropic difference scattering can be calculated as
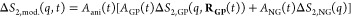
6with the amplitude *A*_ani_(*t*) accounting for rotational dephasing (see Figure S18) and with Δ*S*_2,GP_ and Δ*S*_2,NG_ representing the anisotropic difference scattering from the GP and NG species, respectively. Using this approach of modeling the time-dependent difference scattering curves, the structural refinement is performed for each time step in the data set, optimizing the parameter vector  utilizing the unconstrained minimization function (fminunc) in MATLAB R2022 (Mathworks). Due to the large number of free parameters, we implemented the optimization of the parameter vector in a regularized χ^2^ minimization framework, where a regularization penalty *f* was added for jumps in **x** from one time point to the next (for details, see the SI), which is illustrated in Figure S12 comparing the refined distances for the time-dependent structure of I_3_^–^ in acetonitrile with and without regularization. The χ^2^ includes both the isotropic and anisotropic contributions to the total difference scattering signal:
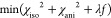
7with the regularization penalty *f*, the regularization factor λ, and
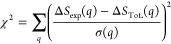
8This constrained minimization of χ^2^(**x**) was performed individually for each time point and repeated 10 times per time point with different starting guesses for **x** to avoid local minima. The starting values were chosen randomly in a range of physically feasible values. The uncertainty of the optimized values from the structural refinement was determined in a separate calculation determining the sensitivity of χ^2^ + *f* for changes of the parameter. For details, see the SI.

## Results

**Figure 3:**
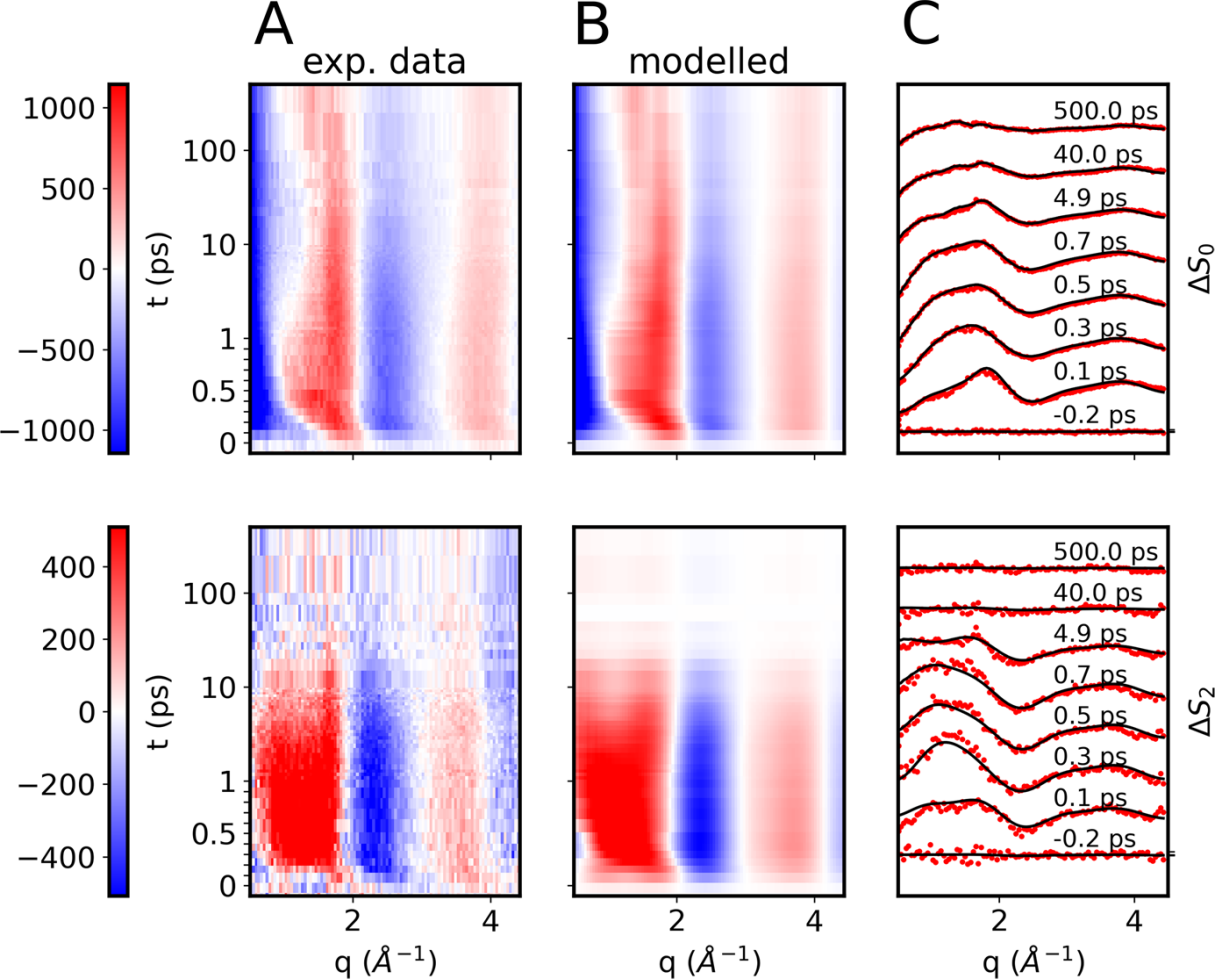
Comparison of modeled and experimental data for I_3_^–^ in acetonitrile. The top row shows data for isotropic (Δ*S*_0_) and the bottom row, for anisotropic (Δ*S*_2_). (A) Experimental difference scattering. (B) Modeled data. (C) Both model (black solid lines) and experimental data (red dots) for selected time points.

Figure 1B shows the time-dependent isotropic difference scattering signals, Δ*S*_0_(*q*, *t*), in the four different solvents (acetonitrile, water, ethanol, and methanol). The data is presented on a linear time scale for the first picosecond to highlight the initial dynamics and on a logarithmic scale for longer pump–probe time delays. Immediately after the photoexcitation at *t* = 0, we observe an oscillatory signal along the *q*-axis with positive peaks around 2 and 4 Å^–1^. On a time scale of 500 fs, the peak at 2 Å^–1^ evolves toward 1.2 Å^–1^ in all solvents. As detailed in the preceding section, these observed time-resolved difference signals were modeled including two laser-induced species, a geminate pair (GP), and a solvent separated pair (NG) including the associated changes in the solvation cage, as well as contributions from bulk solvent heating. Figure 3A shows the experimental data Δ*S*_0_(*q*, *t*) (top row) and Δ*S*_2_(*q*, *t*) (bottom row) for the acetonitrile data set only, with Figure 3B showing the corresponding best-fit modeled data. Figure 3C shows the data and fit for selected time points. The model captures very well both the short- and long-term dynamics of the experimental data in the full *q*-range, and similarly good agreement between experimental and modeled data was achieved for the other three solvents investigated. Figures S10 to S12 show these results.

**Figure 4:**
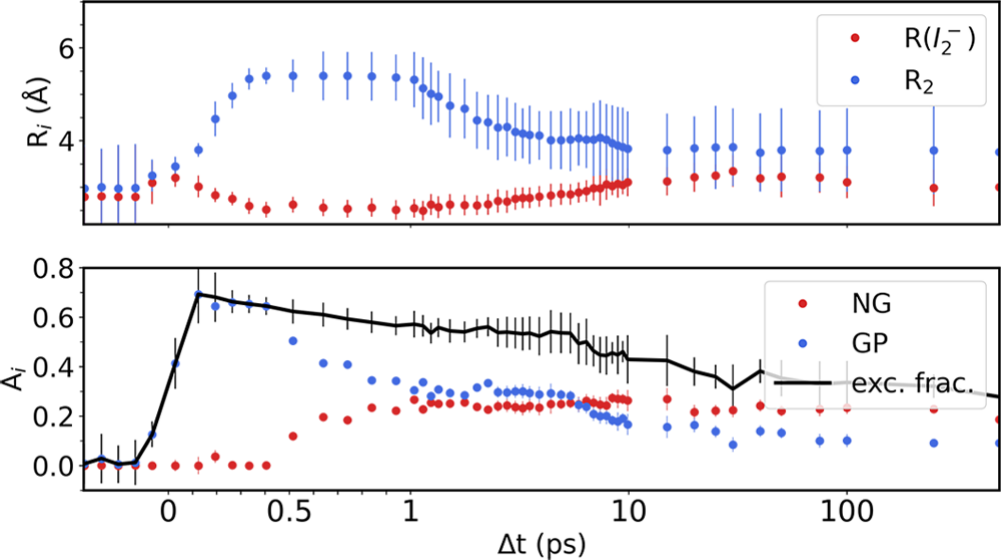
Optimized parameters from the structural refinement for I_3_^–^ in acetonitrile. The two panels present the optimized structural parameters (top) and population dynamics (bottom). A linear scale is used for the first 1 ps to highlight the fastest dynamics; later time points are presented on a logarithmic scale. Amplitudes as defined in eq 5.

Turning next to the results of the time-resolved structural analysis for the data of triiodide in acetonitrile. Figure 4 (top) shows the optimized I–I distances  and as a function of time and the relative concentrations of the GP and NP species as well as the overall scaling *A*_iso_(*t*) (bottom). A fast increase of the interfragment distance  from 3 to 5.5 Å is observed within the first 500 fs where  stagnates for 2 ps, followed by equilibration at a distance of 4 Å. As discussed in further detail below, we tentatively associate these dynamics with ballistic fragment dissociation until the I fragment meets the solvent cage after which some I fragments escape and form the nongeminate population. The interatomic distance of the I_2_^–^ fragment (*R*(I_2_^–^), red in Figure 4, top) shows an initial increase followed by a slight bond shortening. On a time scale of 5 to 10 ps, *R*(I_2_^–^) is observed to reach a new equilibrium at a distance of about 3 Å. We note that the error bars on  increase significantly on the 0.5 to 1 ps time scale (for an approach of error estimation; see the SI), an observation we ascribe to the relative simplicity of how the interfragment distance of the GP species is included in the modeling.

**Figure 5:**
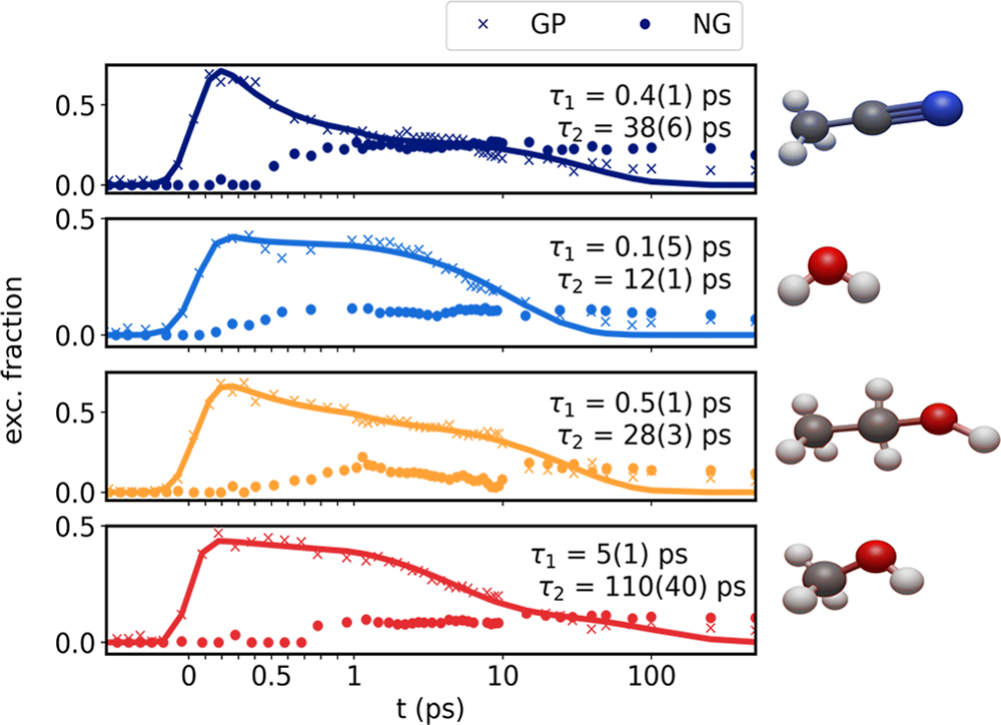
Time dependent amplitudes of the GP (crosses) and NG (filled dots) for the four solvents (from top to bottom: acetonitrile, water, ethanol, methanol). The GP amplitudes are modeled with a biexponential decay, presented as a solid line. The lifetimes of the GP in the different solvents are given in the plot.

Figure 4, bottom, shows the time-dependent amplitude of the total signal *A*_iso_(*t*) and of the GP and NG species as described by the *A*_GP_ fit parameter. The total and GP population increases immediately after laser excitation, reaching its maximum after 200 fs whereas the NG population rises more gradually and reaches a plateau around 1 ps. This is approximately in the same time range as  has reached its maximum and begins to decrease toward the equilibrium value as discussed further below. On time scales exceeding 1 ps, the geminate population is decreasing, while the NG population stays almost constant for the time scales observed here. From the observed population dynamics, the cage escape probability discussed in the Introduction can be estimated by calculating the ratio between the maximum excitation fraction (*A*_iso_) and the average *A*_NG_ in the range from 1 to 2 ps. For acetonitrile, this procedure yields a cage escape probability *P*_e_ = 0.38. Figure 5, top, shows the GP population for acetonitrile (blue crosses), modeled by a biexponential decay convoluted with a Gaussian function (blue solid line). The GP population decay is observed to be well captured by this biexponential fit, with the two time constants being 0.4(1) ps and 38(6) ps. The population of NG (filled dots) survives for the time scales investigated here. The lower panels show the same results for the population dynamics in the other three solvents along with similar biexponential fits to the GP population dynamics.

Comparing the results for the different solvents, we observe that the population of the GP (crosses) increases immediately after laser excitation for all solvents, and the subsequent dynamics are observed to be well captured by the above-mentioned double-exponential model, here shown with solid lines. The short (τ_1_) and longer (τ_2_) time constants range from τ_1_ = 0.1 to 5 ps and τ_2_ = 10 to 110 ps. The ratio between the amplitudes of the two GP lifetimes (*A*_1_/(*A*_1_ + *A*_2_); see eq S12) is 5% in water, 30% in methanol, 40% in ethanol, and 70% in acetonitrile. Similar to the population dynamics observed in acetonitrile, in all solvents, the population of the NG species (filled dots) reaches a plateau within 0.5 to 1 ps and decays on time scales spanning longer than the presented time range. The cage escape probability ranges from *P*_e_ = 0.19 for methanol over *P*_e_ = 0.24 for ethanol and *P*_e_ = 0.27 for water to *P*_e_ = 0.38 for acetonitrile.

**Figure 6:**
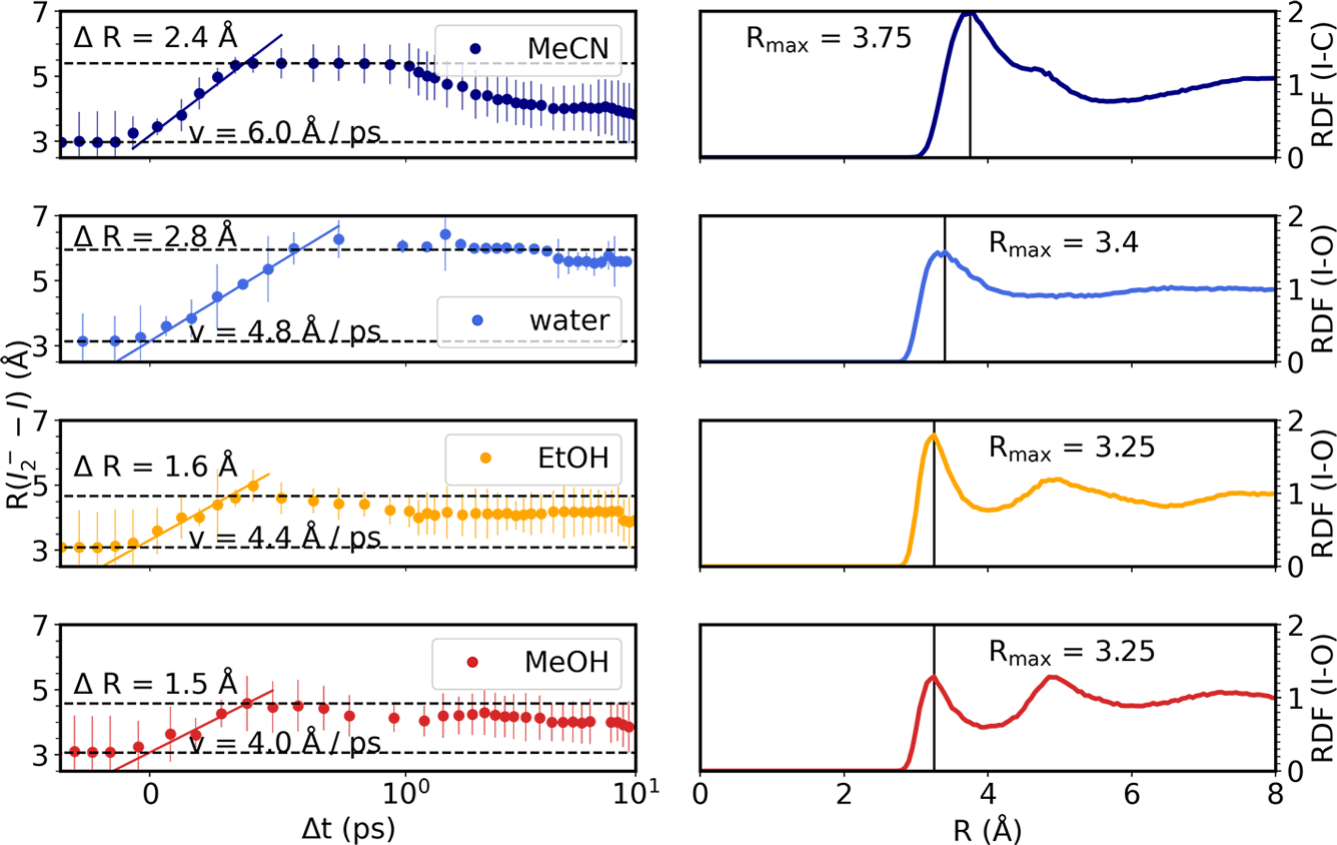
Left: Evolution of the distance , illustrating the bond dissociation. From the initial increase, the speed of dissociation, *v*_d_, is estimated (for ethanol, the data point at *t* = 0.4 ps was excluded from the speed estimate). Right: Radial distribution functions of the solvent O atoms (C for acetonitrile) around the I atoms in their ground state I_3_^–^ structure. Solvents from top to bottom: acetonitrile, water, ethanol, and methanol.

The left column of Figure 6 shows the interatomic distances  as a function of time in all solvents. This can be used to estimate the speed of dissociation, *v*_d_, via . To estimate *v*_d_, we assume a linear increase in interfragment distance from *t* = 0 and until the fragments of the GP populations show the largest interfragment distance , i.e., when the fragment is stopped by the solvent cage molecules. The solid lines in Figure 6, left, indicate these dynamics. The speed of fragment dissociation estimated using this approach takes values of 4.0 Å ps^–1^ for methanol, 4.7 Å ps^–1^ for ethanol, 4.8 Å ps^–1^ for water, and 6.0 Å ps^–1^ in acetonitrile.

The estimated change in  until impact on the solvent cage ranges from 1.5 Å for methanol over 1.9 Å for ethanol and 2.4 Å for acetonitrile to 2.8 Å for water. For comparison, Figure 6, right, shows the radial distribution functions *g*_I–O/C_ for the ground state structure of I_3_^–^ in the four solvents, with the first peak at *R*_max_ indicating the average distance between the terminal I atoms and the nearest non-hydrogen solvent atoms. This first peak can be used as an estimate of the solvent cage size as seen from the viewpoint of a dissociating I fragment. We generally observe larger cage sizes from the RDFs but find good agreement of the relative cage sizes between the  indicated in the left-hand column and this measure for acetonitrile, ethanol, and methanol. For water, the cage size from  is larger than for the other solvents unlike the estimate from *g*_I–O_.

The final parameter included in the refinement of the structural models of dissociation (Figure 2) is the I–I–I angle, α. The changes in α encode the rotation of the I_2_^–^ fragment after bond breakage. Figure S18 shows α(*t*) for the four solvents, and as for the linear displacement, we find a fast change followed by a plateau, which we again interpret as arising from initial ballistic motion followed by a collision with the surrounding solvent molecules of the cage structure. Although there is significant uncertainty in this parameter, the angular speed ω after dissociation can be estimated with the same approach as used for the linear dissociation of the I fragment, and we find ω in the range from ω = 3 to 4.5 rad ps^–1^. Based on the estimates of the linear and rotational speeds, the energy partitioning between translational and rotational degrees of freedom after dissociation can be estimated. As Table 2 shows, the amount of energy released into the rotation is significantly higher (by a factor of 3–5) than energy released into the translation, and this trend is similar for all solvents. For the total kinetic energy after dissociation, we find this to be in the range of 20 to 50 kJ mol^–1^, compared to the excitation energy of a 400 nm photon of ∼300 kJ mol^–1^.

**Table 2:** Energy Partitioning into Rotational and Translational Kinetic Energy as Well as the Temperature Increase for All Solvents

	*v*_diss_ (Å ps^–1^)	*E*_trans_ (kJ mol^–1^)	ω (rad ps^–1^)	*E*_rot_ (kJ mol^–1^)
acetonitrile	6.0(4)	15.2	5(2)	50(20)
water	4.8(2)	9.8	4(1)	40(10)
ethanol	4.4(5)	8.2	4(3)	38(30)
methanol	4.0(5)	6.8	3(1)	22(7)

## Discussion

**Figure 7:**
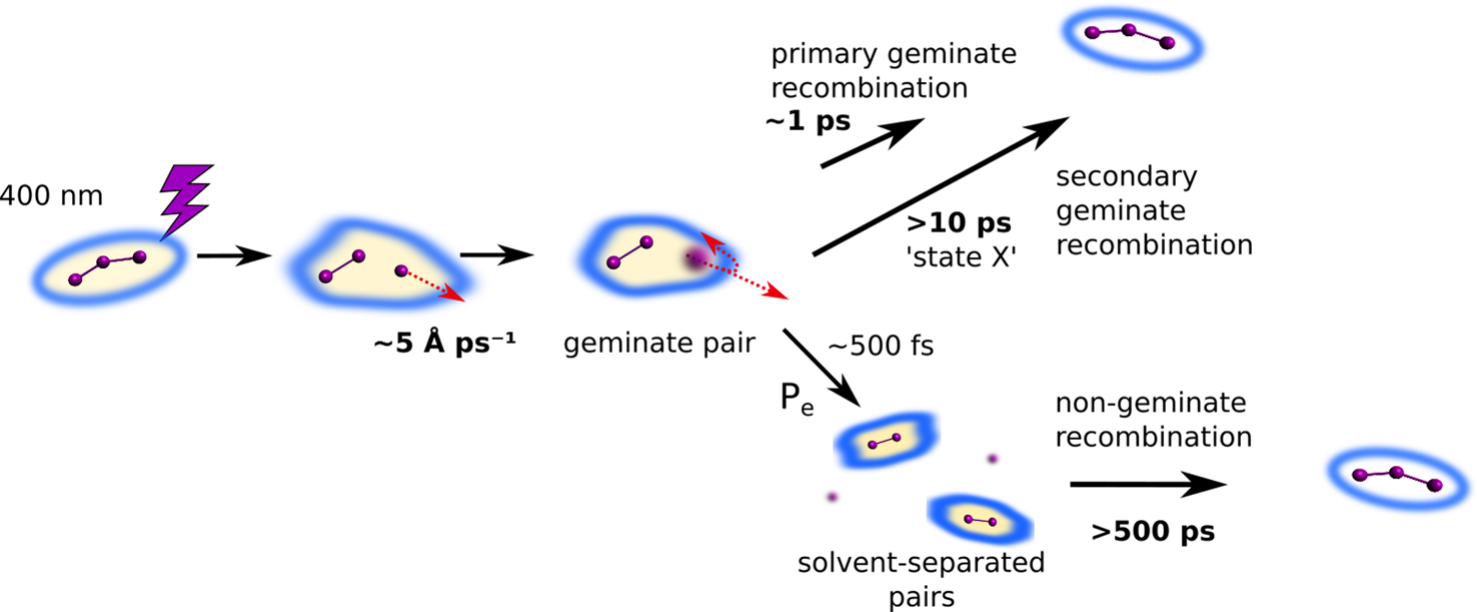
Scheme of the reaction mechanism of the photodissocation and recombination of I_3_^–^.

In the preceding sections, the results of a time-resolved analysis of the dynamics following photodissociation of I_3_^–^ were presented. The model included both structural parameters and a description of population dynamics. The changes in the solvent cage were included by implementing a library of solvent structures around 17,336 different solute structures. From the optimized model parameters (**x**_opt_(*t*)) of the structural refinement in the different solvents, we can conclude that the qualitative mechanisms of bond dissociation followed by geminate and nongeminate recombination is the same for all solvents. This model is in agreement with previous results from spectroscopy,^38,39,50^ and Figure 7 summarizes the model. As discussed in the Introduction, spectroscopy experiments have indicated the presence of a species “X” with a lifetime in the 10s of picoseconds range.^37,38,50^ From the present results, we propose to identify this species as an  geminate pair recombining via secondary geminate recombination on a solvent-dependent time scale of 12 to 110 ps. The long-lived geminate pair is reminiscent of secondary geminate recombination observed for mercury halides, however only showing one decay lifetime.^33^

The results of the structural analysis presented in Figures 4 and 6 suggest an average distance between the I and the center of the I_2_^–^ fragment of 4 to 6 Å for the contact pair. This is in good agreement with the I–I distance found in the CH_2_I_2_ photodissociation study mentioned above although somewhat shorter than the ∼6 Å reported in a previous study for the I_3_^–^ system.^43^ We note that in the mentioned study for the I_3_^–^ system only one species was included in the modeling, unlike the explicit inclusion of geminate and nongeminate pair presented here. Additionally, the increase of error bars on the structural parameters after *t* > 1 ps indicates a level of structural disorder larger than what is included in the present model. From the same structural analysis, we find a prompt increase of the I_2_^–^ bond length immediately after the dissociation event followed by a decrease and subsequent equilibration (>10 ps) to a bond length of about 3 Å in all solvents (Figures 4 and S18). In line with the recent results presented in the previous I_3_^–^ study,^43^ this short-lived bond length increase is assigned to the I_2_^–^ fragment being created in a vibrationally hot state.

As Figure 3 shows, the difference signals calculated based on the model depicted in Figure 7 reproduce the experimental data very well for I_3_^–^ in acetonitrile. Figures S10–S12 show that this is also case for water, methanol, and ethanol. However, differences in structural dynamics and population kinetics between the different solvents are observed. An example of this is shown in Figure 6, left, where for acetonitrile the dissociating I fragment travels a distance of ∼2.4 Å at a speed of 6 Å ps^–1^ following the initiation of the reaction before coming to a halt. For methanol and ethanol, the I fragments reach a lower average speed of less than 5 Å ps^–1^ and the travel distance is about 0.5 Å to 1 Å shorter, observations we tentatively assign to the smaller solvent cages observed for the protic solvents as indicated by the radial distribution functions shown in Figure 6, right. Interestingly, while the average speed in water is similar to the alcohols, we observe a larger travel distance for water (2.8 Å) than for any of the other solvents, not in agreement with the cage size estimate from the radial distribution function. The peak indicating the first solvent shell in the RDF is however less pronounced for water than for the other solvents, indicating a less defined solvent shell which could explain the larger travel distance. The generally smaller travel distances from the refined  compared to the solvent cage size estimates from RDFs can be attributed to neglecting the van der Waals radii of the fragment and solvent atoms in the estimate from RDFs. Figure 6 shows the RDFs of the different solvents around a ground state structure of I_3_^–^. A comparison of RDFs around different structures of I_3_^–^ throughout its dissociation and recombination is presented in Figure S8.

Unlike previous studies, the cage escape probability is directly observed by the population of two different species included in the model, the geminate and nongeminate pair. The cage escape probability is observed to be largest for acetonitrile (∼0.38) and ranges from 0.19 to 0.27 for the H-bonded solvents in qualitative agreement with previous estimates from spectroscopy (0.08 to 0.26,^37^ <0.3 to 0.5^40^). However, among the H-bonded solvents, no clear trend as a function of molecular weight could be observed due to the high uncertainty of the estimates. Generally, we observe slightly lower cage escape probability than in a previous study by Gershgoren et al.,^40^ which can be attributed to the longer excitation wavelength used here (400 nm vs 308 nm), leading to less kinetic energy of the dissociating fragments and accordingly lower cage escape.

We have estimated the energy partitioning from the translational and rotational speed of the radicals (Table 2). In agreement with our study on CH_2_I_2_,^22^ we find that a small fraction of the photon energy (400 nm) is transferred into the dissociating fragments as kinetic energy. This is reasonable considering that most of the energy is contained as potential energy in the electronic state. Depending on the solvent, 2–5% of the energy is used for translation and about 7–18%, for rotation of the I_2_^–^ fragment. The fragments have a significantly higher relative velocity in acetonitrile compared to the three protic solvents. It is tempting to simply correlate the energy partitioning with the ground state structure. A more bent structure is expected to lead to higher rotational excitation of the diiodide fragment on the cost of translation and vice versa. However, the rotational excitation in acetonitrile, despite the only slightly bent ground state structure of triiodide in this solvent,^10,57^ indicates that this picture is too simplistic. Instead, we propose that the partitioning rather depends on the amount of photoexcitation energy channelled into the bending modes compared to the dissociating stretching mode of the molecule. Triiodide has dominant symmetric and asymmetric stretching modes, as well as a bending modes at 114, 145, and 59 cm^–1^, respectively.^38^ From this, an alternative explanation of the energy partitioning follows where the coupling of the electronic transition to the stretching vibration is approximately twice as high in acetonitrile compared to the protic solvents, and a strong excitation of the bending vibration occurs in all solvents. As a final possible explanation, we propose that it may also be that the energy partitioning across rotational and translational movements is critically controlled by the oscillatory acceleration of the atoms along the modes. In this model, the acceleration of the atoms at the time point where bond breaking occurs will determine the partitioning.

In terms of population dynamics, we find that the nongeminate population fraction starts to increase on a time scale of 0.5 to 1 ps, concomitant with the time needed for the dissociating I fragment to reach the “wall” of the solvation cage and subsequently escape with the probability as described above. For the population fraction where the I remains trapped in the cage, the geminate pair (GP), Figure 5 shows geminate recombination occurring on two time scales, τ_1_ = 0.1 to 5 ps and τ_2_ = 12 to 110 ps. These time scales qualitatively match lifetimes assigned to geminate recombination and state “X” in previous studies on triiodide^38,39,50,75^ and compare to recombination via primary and secondary geminate recombination observed for ICN and mercury halides.^33,34^ Due to the increasing structural uncertainty as represented by increasing error bars on the refined distances, we can however not unambiguously identify the structural differences between these two recombination pathways with fragments sharing either a joint solvent shell (primary geminate recombination) or a contact pair separated by only few layers of solvent molecules (secondary geminate recombination). The solvent dependence of τ_2_ follows trends previously observed in spectroscopy with a relatively short lifetime in water (12 ps) and longer lifetimes for acetonitrile and ethanol.^50^ Differences between the lifetimes observed here and in previous studies may be caused by limited spectral and temporal windows in our and previous studies possibly affecting the kinetics.^39^ For the nongeminate population, the recombination is much slower, on the order of hundreds of picoseconds to nanoseconds in all solvents as previously observed.^10,37−39^ Lifetimes on nanosecond time scales suggest this pathway is nongeminate recombination and not a biexponential behavior of SGR as observed for mercury halides.^33^ This is confirmed by our structural refinement, identifying the structure of this species as solvent-separated pair.

A limiting factor of the present study is the comparatively low signal-to-noise ratio and limited *q*-space coverage. In combination with a large number of free parameters in the analysis (parameter vector **x**), this leads to large confidence intervals on some of the parameters. However, with the continued improvements in XFEL performance as evidenced by the recent study of Heo et al.,^43^ this concern is diminished and the overall experimental and analysis approach developed, in both that study and the present work, should be directly applicable for further studies with significantly higher temporal and spatial resolution; the latter will be achieved by higher X-ray energies in the future which will enable higher *q*-space coverage.

## Conclusions

We have presented a model which provides a comprehensive picture of the structural dynamics of the photodissociation and recombination of triiodide in solution. This model has been applied to time-resolved X-ray solution scattering data for I_3_^–^ in four different solvents and very well reproduces the experimental results. A key result is the identification of a long-lived  geminate pair as the “State X” previously suggested on the basis of spectroscopic data. Further, the direct structural information available from X-ray scattering reveals a mechanistic picture of bond dissociation followed by ballistic movement and finally an encounter with the caging solvent molecules. These conclusions are developed and rationalized on the basis of comparison with results from MD modeling. The presented results give direct structural insight into the solvent–solute interactions by determining the solvent cage size and observation of the influence of hydrogen bonds in the solvent on the cage escape probability. With the advances at XFEL facilities, we expect the possibility for more detailed insights in the future.

## Supporting Information Available

Additional details on experimental methods, data reduction, molecular dynamics simulations, and data analysis (PDF)
