# Supplementary material for: Solvent-Dependent Structural Dynamics in the Ultrafast Photodissociation Reaction of Triiodide Observed with Time-Resolved X-ray Solution Scattering
Source: J Am Chem Soc. 2023 May 10;145(29):15754–65. doi: 10.1021/jacs.3c00484 (PMC10375522; doi:10.1021/jacs.3c00484)
Supplement: Supplementary file 1 — ja3c00484_si_001.pdf [file ja3c00484_si_001.pdf]

Supporting Information for: Solvent-dependent structural dynamics in the ultrafast photodissociation reaction of triiodide observed with Time-Resolved X-ray Solution Scattering

Amke Nimmrich,[†,△] Matthijs R. Panman,[†] Oskar Berntsson,[†] Elisa Biasin,[‡,∇] Stephan Niebling,[†,††] Jonas Petersson,[¶] Maria Hoernke,[†,‡‡] Alexander Björling,[†] Emil Gustavsson,[†,¶¶] Tim B. van Driel,[‡,§§] Asmus O. Dohn,[‡,§] Mads Laursen,[‡] Diana B. Zederkof,[‡,‖‖] Kensuke Tono,[‖] Tetsuo Katayama,[‖] Shigeki Owada,[⊥] Martin M. Nielsen,[‡] Jan Davidsson [a,¶] Jens Uhlig,[#] Jochen S. Hub,[@,⊥⊥] Kristoffer Haldrup,[‡] and Sebastian Westenhoff[*,†,##]

†*Department of Chemistry and Molecular Biology, University of Gothenburg, Box 462, 40530 Gothenburg, Sweden*

‡*Department of Physics, Technical University of Denmark, DK-2800 Lyngby, Denmark*

¶*Department of Chemistry, Ångström Laboratory, Uppsala University, Box 523, SE75120 Uppsala, Sweden*

§*Faculty of Physical Sciences, University of Iceland, VR-III, 107 Reykjavík, Iceland*

‖*Japan Synchrotron Radiation Research Institute, 1-1-1 Kouto, Sayo-cho, Sayo-gun, Hyogo 679-5198, Japan*

⊥*RIKEN SPring-8 Center, 1-1-1 Kouto, Sayo-cho, Sayo-gun, Hyogo 679-5148, Japan*

#*Department of Chemical Physics, Lund University, Box 124, Lund, Sweden*

@*Georg-August-Universität Göttingen, Institute for Microbiology and Genetics, Justus-von-Liebig-Weg 11, 37077 Göttingen, Germany*

△*Current affiliation: Department of Chemistry, University of Washington, Box 351700, Seattle, Washington 98195, USA*

∇*Current affiliation: Physical Sciences Division, Pacific Northwest National Laboratory, Richland, Washington 99352, USA*

††*Current affiliation: EMBL, Notkestrasse 85, DE22607 Hamburg, Germany*

‡‡*Current affiliation: Chemistry and Pharmacy, Albert-Ludwigs-Universität, Hermann-Herder-Str. 9, 79104 Freiburg i.Br., Germany*

¶¶*Current affiliation: Karolinska Institute/Centre for Structural Systems Biology, Notkestrasse 85, DE22607 Hamburg, Germany*

§§*Current affiliation: LCLS, SLAC National Laboratory, Menlo Park, California 94025, USA*

‖‖*Current affiliation: European XFEL GmbH, Holzkoppel 4, DE22869 Schenefeld, Germany*

⊥⊥*Current affiliation: Theoretical Physics, Saarland University, Campus E2.6, 66126 Saarbrücken, Germany*

E-mail: westenho@chem.gu.se

[a]Sadly, Prof. J. Davidsson passed away on June 24, 2019.

Experimental

Sample Preparation

The triiodide ion (I_3^-) was generated *in situ* by dissolving molecular iodine (I_2, Wako Pure Chemical Industries) and potassium iodiode (KI, Wako Pure Chemical Industries) in the respective solvent in the amounts according to table S1.

Table S1: Sample details.

Sample	Solvent	$c(I_2)$ / mM	c(KI) / mM	preparation method
I_3^-	H_2O	9.250	69.30	sonicated for 60 min
I_3^-	MeOH	9.125	18.25	sonicated for 10 min
I_3^-	EtOH	9.150	19.90	stirred for 1.5 h
I_3^-	MeCN	9.30	18.50	sonicated for 60 min

An excess of KI was used to ensure that the $KI + I_2 \rightleftharpoons I_3^- + K^+$ equilibrium strongly favors the formation of I_3^-. The effective concentration of I_3^- can hence be estimated as $c(I_2)$. The electronic absorption spectra of the I_3^- solutions in Supplementary Table S1 are shown in Supplementary Fig. S1. Methanol (MeOH), ethanol (EtOH), and acetonitrile (MeCN), were HPLC grade and

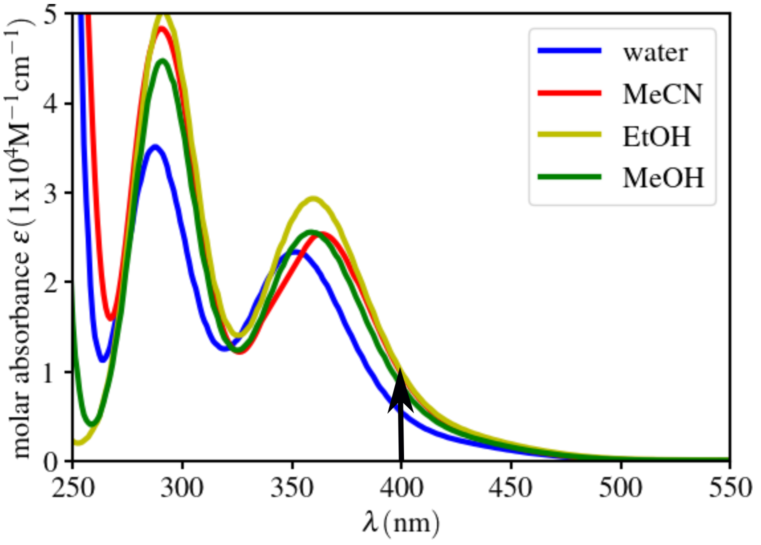

Figure S1: Electronic absorption spectra of 0.4 mM I_3^- in the various solvents. The absorption spectra were measured in a 1 mm pathlength cuvette. The black arrow denotes the wavelength of the 400 nm pump pulse.

obtained from Wako Pure Chemical Industries. H_2O was milliQ grade. The Fast Yellow dye (2-amino-5-[(*E*)-(4-sulfophenyl)diazenyl]benzenesulfonic acid) was obtained from Sigma-Aldrich.[1] All chemicals were used without further purification. All experiments were performed at room temperature.

Time-Resolved X-ray Solution Scattering Experiment

The pump pulse was generated by second-harmonic generation (SHG) of the 800 nm fundamental from a Legend Elite (Coherent) and an in-house-designed multi-pass amplifier (~15 mJ pulse energy, <40 fs FWHM pulse duration). The CPA system is synchronized to the XFEL operating frequency and is described elsewhere.[2] The optical pump pulse with a fluence of 80 mJ cm^{-2} (450 × 450 µm FWHM beam dimension, 400 nm, <100 fs FWHM, 450 µJ pulse energy) and the unfocused XFEL beam (300 µm FWHM diameter, 11.98 ± 0.028 keV, 10 fs FWHM, 30 Hz repetition rate) were spatially overlapped in a near-colinear geometry on a liquid-sheet jet (100 µm thick) and propagated horizontally with respect to the laser table. Overlap (300 µm to 600 µm offset below the nozzle outlet) was assured using two remote-controlled microscope cameras. The distance between the sample jet and the detector (octal MPCCD)[3] was kept at 62 mm throughout the experiment. The detector was vertically offset from the center by 80 mm. A beam stop was positioned between sample and a 50 µm thick Kapton window. With the described parameters a momentum transfer range of $q = 0.53\,\text{Å}^{-1}$ to $5.98\,\text{Å}^{-1}$ was achieved. The sample delivery was mounted such that the X-ray beam was normal to the plane of the liquid jet. The chamber was kept under He atmosphere.

Determination of the Instrument Response Function

The time resolution of the experiment was limited by the pulse widths of optical pump- and X-ray probe pulses and their velocity mismatch when passing the liquid jet. To determine the actual time-resolution of the experiment, the Instrument Response Function (IRF) was determined by a least square fit of the integrated signal in the range of $q = 1.5\,\text{Å}^{-1}$ to $2.5\,\text{Å}^{-1}$ against the following

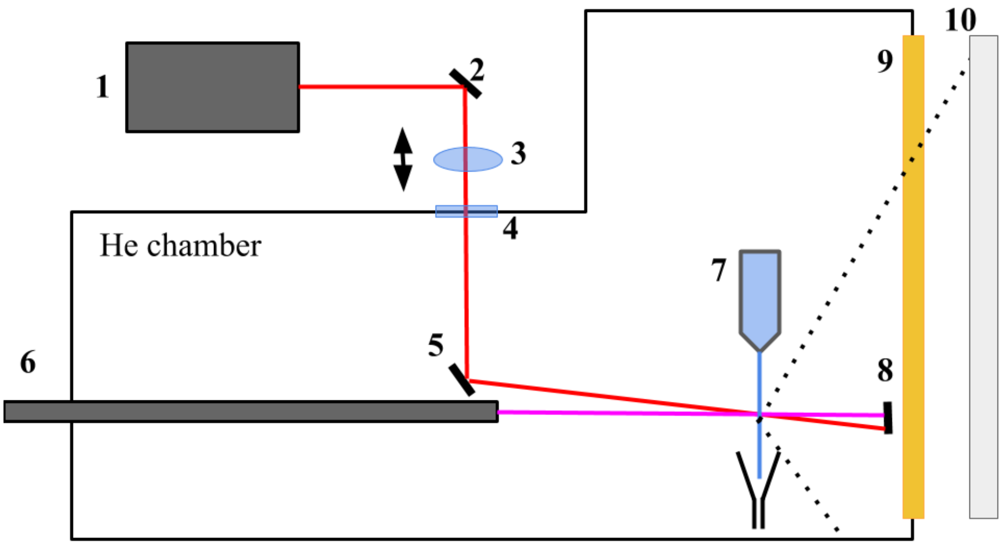

Figure S2: **1**: Laser and SHG of the 400 nm light. **2**: Mirror. **3**: Adjustable CaF_2. **4**: µm-thick CaF_2 window. **5**: Remote-controlled adjustable mirror. **6**: X-ray flight tube. **7**: Sample delivery system. **8**: Beamstop. **9**: 50 µm thick kapton window. The components are not to scale.

model:

$$f(t) = 0.5 \cdot \left(A \cdot \exp\left(\frac{(\sigma^2 - 2 \cdot (t - t_0) \cdot \tau)}{(2 \cdot \tau^2)} \right) \cdot \mathrm{erfc}\left(\frac{(\sigma^2 - (t - t_0) \cdot \tau)}{(\sqrt{2} \cdot \sigma \cdot \tau)} \right) \right) + O\,, \tag{S1}$$

The model and experimental data are presented in Fig. S3 and the resulting values for σ and t_0 are presented in Tab. S2. The presented data are corrected for the t_0 offset.

Table S2: Values for σ and t_0 obtained from fitting the instrument response function for the different solvents.The experimenntal data were corrected for the t_0 offset determined from the IRF.

	water	acetonitrile	ethanol	methanol
σ (ps)	0.0756	0.0684	0.0623	0.0601
FWHM (ps)	0.151	0.136	0.124	0.12

Data Reduction

The measured scattering patterns were corrected for background, solid angle coverage, X-ray polarization and common mode fluctuations.[1,4] The sample-detector distance calibration was per-

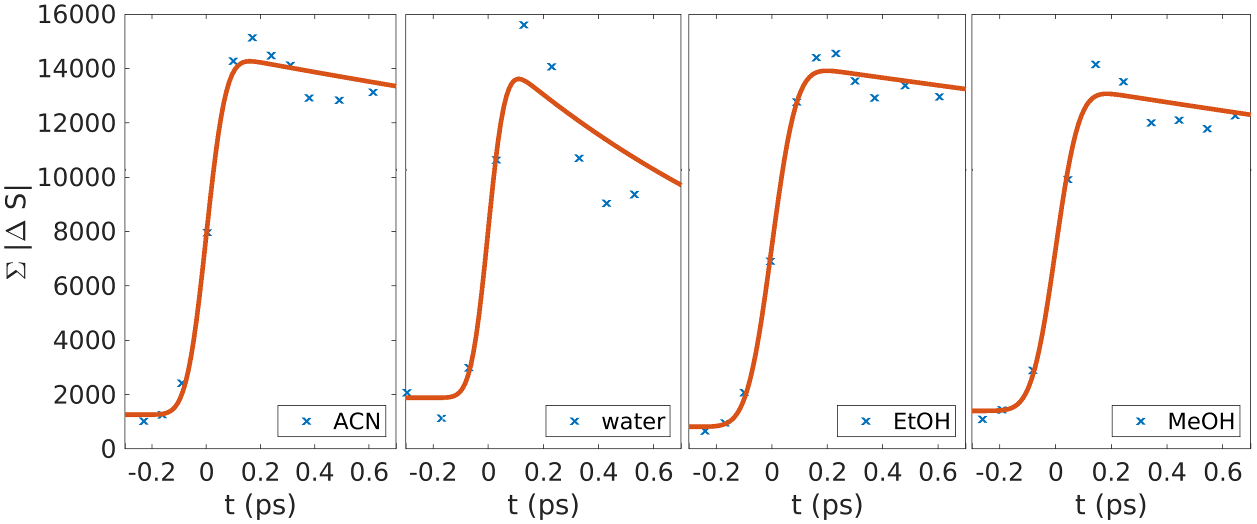

Figure S3: Instrument response function fitted to the integrated absolute signal in the range of $q = 1.5\,\text{Å}^{-1}$ to $2.5\,\text{Å}^{-1}$ for all solvents.

formed by comparison of the azimuthally integrated signal with the scattering signal of pure solvent (water, acetonitrile, ethanol or methanol respectively).[4,5] All scattering curves were scaled to the signal from one liquid unit cell known from reference data in order to obtain signals in units of electron units per solute molecule (e.u. molec.$^{-1}$). Fig. S4 shows the scaled experimental absolute scattering curves (blue) to reference curves (red). The reference curves include coherent and incoherent contributions to the solvent scattering, and the coherent scattering from the solvated I_3^-, as well as scattering from the excess $I^- + K^+$. Scaling was optimised by minimising the difference between experimental and scattering curves in the q-range $1.2\,\text{Å}^{-1}$ to $2\,\text{Å}^{-1}$.

To measure difference scattering curves, every seventh X-ray shot a scattering pattern without laser excitation was recorded. The nearest ten 'dark' scattering patterns to a 'light' measurement were then averaged and subtracted. Binning of scattering curves for different timepoints was performed differently for early and late times. For <2 ps the timing tool was used to determine the time delay with 10 fs (FWHM) accuracy.[6] After time sorting these scattering patterns were then binned where each bin contained ∼7500 curves. At late times (>2 ps) scattering curves at selected time points up to 500 ps were measured where the bins cover a wider time range than for the early delay times.

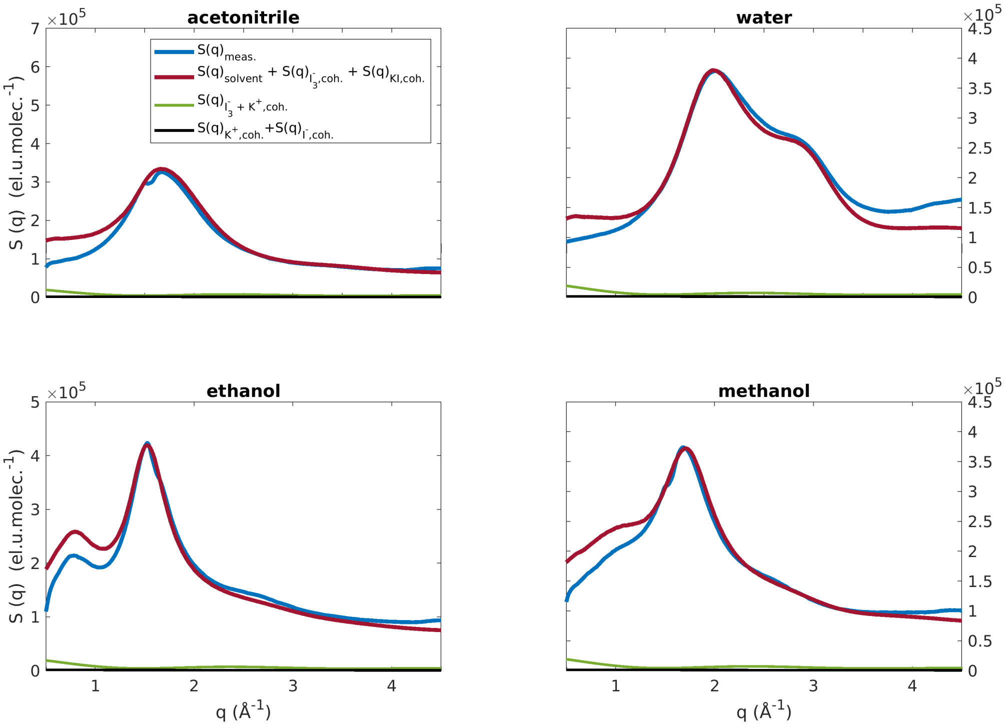

Figure S4: Scaling of the absolute scattering curves in the different solvents to the respective LUC.

Power titrations were performed for the different solvents to make sure there are no significant contributions of multiphoton excitation. Difference scattering was recorded at a time delay of 10 ps for a range of excitation powers between 10 µJ to 600 µJ with 2000 shots per scan. The results are presented below for MeOH and EtOH. The power used in our experiment is indicated with a red line (450 µJ).

Molecular Dynamics simulations

The MD simulations for generating the RDF library for 17,336 $I_3{}^-$ structures were set up as follows: Three iodine atoms with their arrangement described by the structural paramters **R** were placed into a cuboid simulation box, keeping a distance of 1.6 nm between iodine and the box boundary in water, and a distance of 1.7 nm between iodine and the box boundary in all other solvents. The solvation was modelled by placing the solute molecule in a pre-equilibrated simulation box of water, acetonitrile, ethanol or methanol, respectively. Lennard-Jones parameters

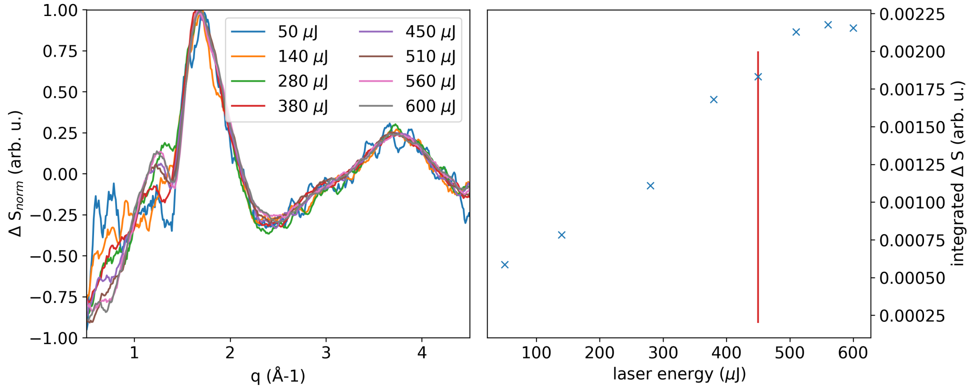

Figure S5: Power titration in ethanol. On the left normalised difference scattering curves measured at different excitation powers are presented. On the right the integrated intensity of the unnormalised curves.

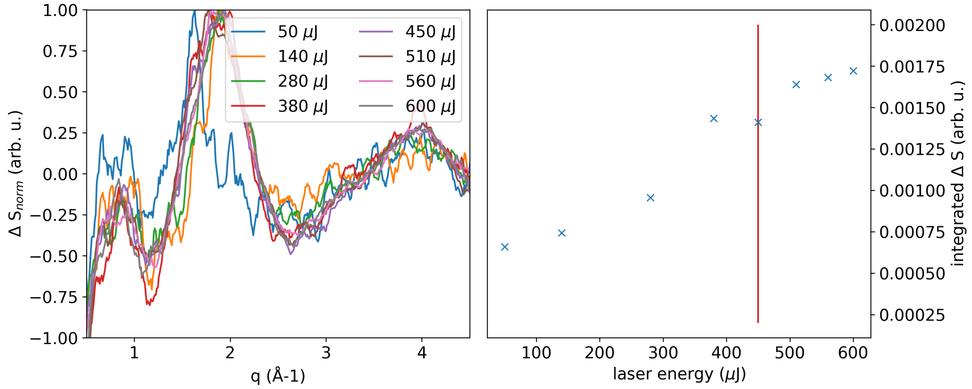

Figure S6: Power titration in methanol. On the left normalised difference scattering curves measured at different excitation powers are presented. On the right the integrated intensity of the unnormalised curves.

for iodine atoms were set to $\sigma = 0.38\,\mathrm{nm}$ and $\varepsilon = 2.092\,\mathrm{kJ\,mol^{-1}\,nm^{-2}}$, taken from the General Amber Force Field (GAFF).[7] The partial charges of the three iodine atoms were adapted from the heuristic model by Benjamin and Ruhman.[8] Accordingly, the partial charges of I_3^- are:

$$q_1 = \frac{+0.25}{1+\zeta\rho^2} - 0.5 \tag{S2}$$

$$q_2 = -1 - q_1 - q_3 \tag{S3}$$

$$q_3 = \frac{-0.25}{1+\zeta\rho^2} \tag{S4}$$

where $\rho = R^2_{I_2^- - I} - R^2_{I_2^-}$ and $\zeta = 0.2$ Å^{-4}. This partial charge model leads to $(q_1, q_2, q_3) = (-0.25, -0.5, -0.25)$ for I_3^- and continuously interpolates to $(q_1, q_2, q_3) \approx (-0.5, -0.5, 0)$ as the third iodine atoms dissociates (with $R_{I_2^- - I} > R_{I_2^-}$). Parameters for acetonitrile, ethanol and methanol were taken from the GAFF topologies deposited at `virtualchemistry.org`.[9] Water was modelled with the SPCE model.[10] The energy of each system was minimised, and each system of the 17,336 structures was simulated for 1 ns. During the simulation, the positions of the iodine atoms were frozen, such that the pre-selected arrangement **R** was maintained.

The simulations were carried out with the GROMACS simulation software, version 4.68.[11] The temperature was fixed at 293.15 K using a stochastic dynamics integration scheme[12] (with a time constant $\tau = 0.5\,\mathrm{ps^{-1}}$), and the pressure was kept at 1 bar using the weak coupling scheme[13] (with a friction constant $\tau = 0.5\,\mathrm{ps^{-1}}$). Bond lengths of the solvent were constrained using LINCS,[14] allowing a time step of 2 fs. Dispersive interactions and short-range repulsion were described by a Lennard-Jones potential with a cut-off at 1.2 nm. Electrostatic interactions were computed with the particle-mesh Ewald method with a Fourier grid spacing of $0.12\,\mathrm{nm^{-1}}$.[15,16]

Fig. S8 show the RDFs around different structures of I_3^- throughout the dissociation and recombination with the following structure for the ground state (GS), two geminate pairs (GP1 and GP2) and a solvent-separated structure (NG). GS: R_1 = 3.01 Å, R_2 = 2.98 Å, α = 3.002, $GP1 : R_1$ = 2.6 Å, R_2 = 6.5 Å, $\alpha = \pi$, GP2: $R_1 = 3.1$ Å, $R_2 = 3.9$ Å, $\alpha = \pi$, and NG $R_1 = 3.1$ Å, $R_2 = 100$ Å, $\alpha = \pi$. The solvent structure gradually changes from the GS species over the GP species to

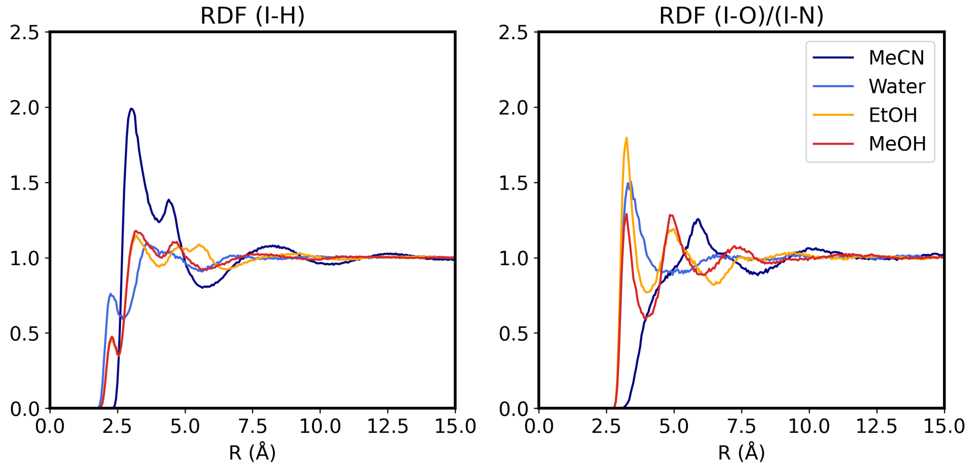

Figure S7: Radial distribution functions between I and H atoms (left), and I and O (right, I–N for MeCN) for a close to GS I_3^- structure, in the different solvents (MeCN, water, EtOH, and MeOH).

the solvent-separated pair. Interestingly, the structure of the GP separated by a layer of solvent molecules (GP2) shows higher similarity to the structure around NG than to the structure around a caged geminate pair. These structures can however still be distinguished by there signatures from the Debye term.

Dynamic vs. equilibrated MD simulations

To investigate the effect of the dynamic solvent rearrangement around the solute compared to the approach using static structures described above, we have performed dynamic MD simulations. This was done examplary for the dissociation of I_3^- in water. The simulations were performed in GROMACS. I_3^- in a water box was first equilibrated, and the excitation simulated by applying an LEPS potential simulating the excited state potential.[17] Simulations were run for 30 starting structures and each for 5 ps and sampled every 0.8 fs.

Fig. S9 shows difference scattering curves calculated for the dynamic MD simulations (top), and for the static solvent response to the above described I_3^- structures below. The ground state structure was determined from the structure before initiating the bond-dissociation. For calculating the corresponding scattering of the static solvent response, for each time point the closest structure in the library of I_3^- structures was identified and scattering for this structure calculated (see above).

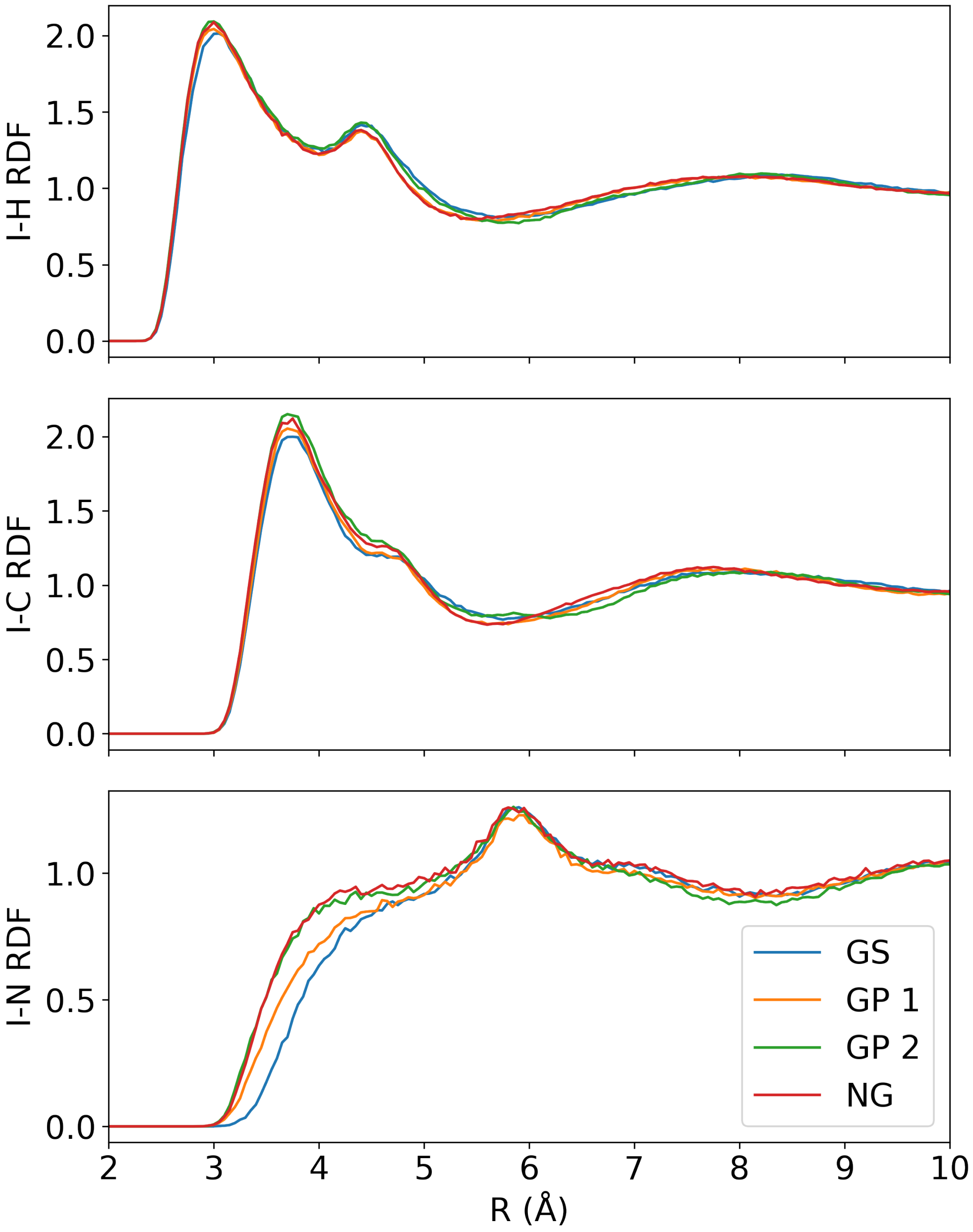

Figure S8: Radial distribution functions between I and H atoms (top), I and C atoms (middle), and I and N (bottom) describing the solvent structure around a GS structure (blue), a caged GP structure (orange), a contact pair structure (green) and a solvent separated pair structure (red).

When comparing the two time-dependent difference scattering curves, we only observe minor differences between scattering calculated from static and dynamic MD simulations. Hence, we used the library of static solvent cages for modelling the difference scattering in the structural refinement. In the case of the photodissociation of I_3^- the strongest contributions to the difference signal are expected from the solute-solute term.

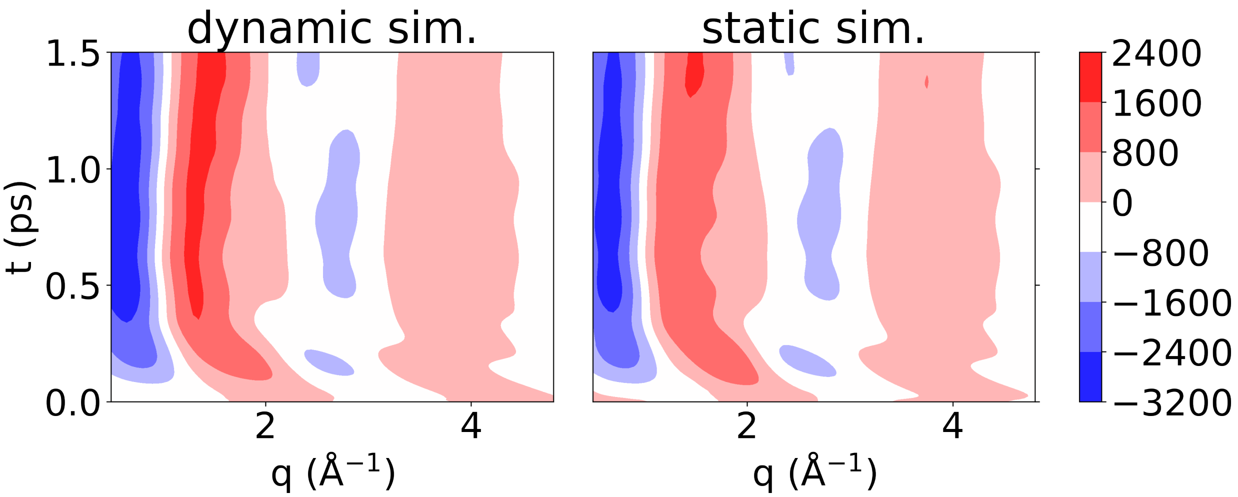

Figure S9: Comparison of difference scattering curves obtained from dynamic MD simulations (top), and difference scattering for the same I_3^- structures using equilibrated solvent (bottom).

Data Analysis

Calculation of contributions to the modelled scattering curves

As described in the main text, we include contributions from a solute-solute term (S_{solute}), and a cage term (S_{cage}) when calculating scattering for a specific solute structure. S_{cage} includes contributions from a solvent-solute cross term (S_c), and a displaced volume term (S_v). Calculation of (S_{solute}) is described in the main text (eqs. 5 and 6). For calculation of S_c we used the RDFs between the I atoms and solvent atoms v, $g_{Iv}(r)$, from the above described MD simulations.

$$S_c(q,R) = 2\int \rho_c(r)\,\mathrm{sinc}(qr)\mathrm{d}r = 2\sum_v F_I F_v \rho_{v,0} N_I 4\pi \int (g_{Iv}(r)-1)r^2\,\mathrm{sinc}(qr)\mathrm{d}r. \quad \text{(S5)}$$

[18,19]

With the respective form factors F_i for solvent (v and solute (I) atoms. In order to calculate the scattering contributions caused by changes in the structure of the solvent cage (S_v), we used the displaced volume approach to model the solvent signal.[20] Dummy atoms described by solvent specific form factors, F_{DV}, are being placed in the position of the iodine atoms. The form factors for these dummy atoms determined from the RDFs:

$$F_{DV}(q) = 4\pi \sum_{v} F_v(q)\rho_{v,0} \int (g_{Iv}(r) - 1) r^2 \operatorname{sinc}(qr) \tag{S6}$$

These form factors are then used to determine the solvent scattering using the Debye equation:

$$S_v(q,R) = \sum_{i} F_{DV,i}^2 + 2 \sum_{i,j>i} F_{DV,i} F_{DV,j} \operatorname{sinc}(q d_{ij}). \tag{S7}$$

To minimise truncation errors from the limited box-size in the MD simulations we applied an exponential damping factor ($\exp(-(\frac{r}{\mu} - 1)^2)$) to the $g_{Iv}(r)$ used for calculation of S_v and S_c for r $\geq \mu$.

The solute contributions to the anisotropic scattering are calculated as:

$$S_{\text{ani}}(q,R) = -c_2(t) \sum_{i,j}^{N} F_i^*(Q) F_j^*(Q) P_2(\cos \zeta_{i,j}(t)) j_2(Q_{i,j}(t)) \tag{S8}$$

with the atomic form factors F_i^* including a term describing positional uncertainty (see main text eq. 5), and the Bessel function.

$$j_2(x) = \left((3/x^2) - 1\right)((\sin x)/x) - 3\left((\cos x/x^2\right) \tag{S9}$$

The second order Legendre polynomial is defined as:

$$P_2(x) = (3x^2 - 1)/2. \tag{S10}$$

The anisotropic difference scattering, ΔS_2, can be calculated as:

$$\Delta S_{ani}(q) = A_{ani}(A_{GP}\Delta S_{ani,GP} + (1 - A_{gem})\Delta S_{ani,NG}) \qquad \text{(S11)}$$

Since the solvent response due to heating is assumed to be predominantly isotropic, no heat response is included in modelling the anisotropic signal.

As discussed in the main text, in this study the ground state structure of triiodide in the different solvents was not optimised but adapted from previous MD studies.[21] Fig. S10 shows fit between experimental and modelled data at a delay time of 500 ps in acetonitrile using the ground state structure used in the refinement ($R_1 = 2.95$ Å, $R_2 = 3.06$ Å, $\alpha = 3.002$), the GS structure published by the Ihee group (middle panel, $R_1 = 3.01$ Å, $R_2 = 2.98$ Å, $\alpha = \pi$)[22] and a more bent, but symmetric structure (lower panel, $R_1 = 3.01$ Å, $R_2 = 2.98$ Å, $\alpha = 2.7$). The comparison shows best agreement with experimental data using the structure by Jena et al.

Regularisation

The large number of parameters optimised in the structural refinement (described by the optimisation vector $\mathbf{x} = [A_{iso}, A_{GP}, A_{heat}, A_{ani}, \mathbf{R_{GP}}]$ with $\mathbf{R_{GP}} = [R(\mathrm{I_2}^-), R_{\mathrm{I_2}^- - \mathrm{I}}, \alpha]$), leads to an increasing risk of finding local minima instead of globally optimal values. Therefore, we implemented a regularised χ^2 minimisation to stabilise the fit. The values to regularise against ($\mathbf{x^0}$) were chosen for the first time point ($R = R_{GS}, A = 0.5, A_{gem} = 1, A_{ani} = 0.1, A_{heat} = 0$), and then regularisation was performed against the optimised value from the previous time point.

$$f = \sum (\mathbf{x} - \mathbf{x^0})^2$$

The amplitude of the heat signal was normalised to make sure all parameters are in the same order of magnitude. The optimal λ parameter was determined using the L-curve approach.[23] The refinement was performed over a range of λ values (from 10^{-4} to 10^4), and the resulting penalties f_{tot} were plotted against χ^2_{tot} with $f_{tot} = \sum_t f$ and $\chi^2_{tot} = \sum_t chi^2$, leading to an L-shaped curve

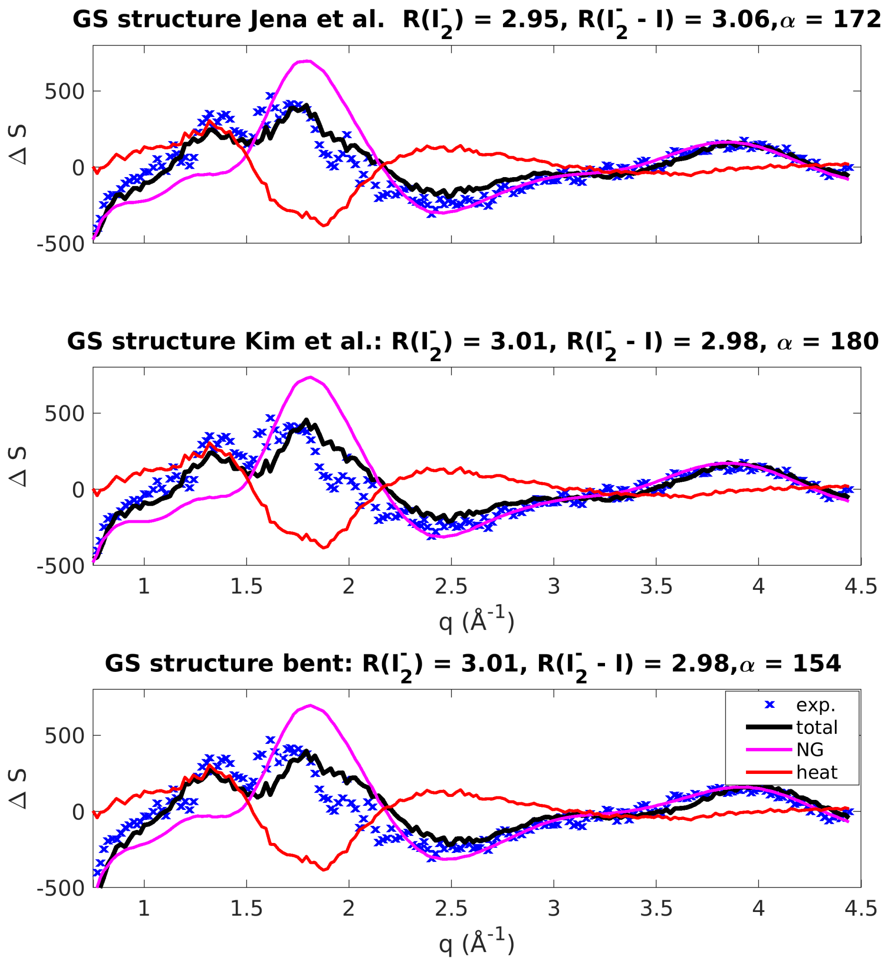

Figure S10: Comparison of fit between experimental (blue crosses) and modelled data (black lines) using two different GS structures - the structures published by Jena et al. as used in the refinement (upper panel), a GS structure published by the Kim et al.[22] (middle panel) and a symmetric but bent (154 deg) structure (lower panel). Plotted are also the contributions from the NG structure (magenta) and heat (red).

(Fig. S11). The optimal λ is then the one corresponding to the point of maximum curvature, in the case of acetonitrile $\lambda \approx 1$ ($\lambda(H_2O) = 2$, λ(EtOH) = 2, λ(MeOH) = 2).

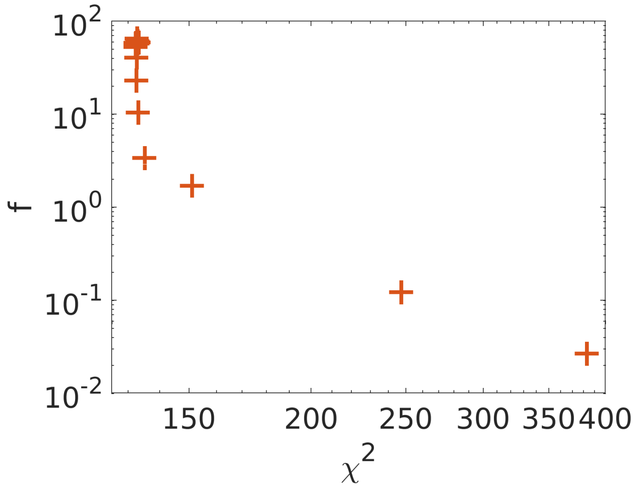

Figure S11: L-curve for determination of the optimal λ parameter for the regularised χ^2 minimisation of the difference scattering data for I_3^- in MeCN. Plotted are the regularisation fine f against the χ^2 for different λ values. The point of maximum curvature at $f \approx 3$ corresponds to $\lambda \approx 1$.

Fig. S12 shows a comparison of results from structural refinement of data for triiodide in acetonitrile with (circles) and without (crosses) regularisation. The overall trend for the structural changes as refined using the two approaches is very similar. However, without regularisation we observe more jumps in distances from time point to time point with in some cases unphysical amplitudes.

Modelled data for all solvents

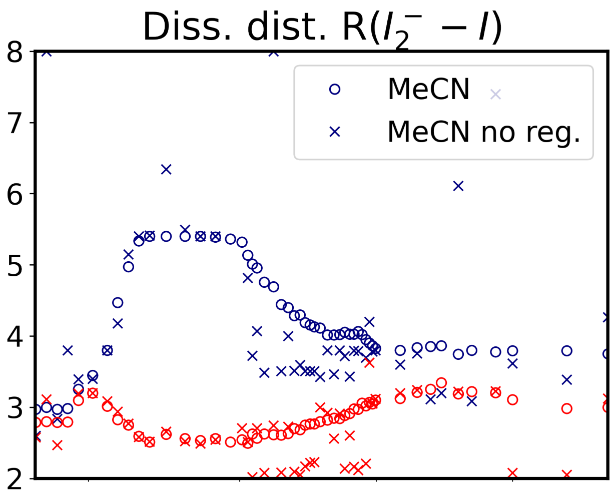

Figure S12: Comparison of structures obtained from refinement with (circles) and without (crosses) regularisation for data of triiodide in acetonitrile.

Heat response of the solvent

Additionally, to the structural signal, the difference signal cause by solvent heating was included in modelling the data. Using reference difference scattering for a 1 K heat increase in the different solvents,[5] this amplitude could be used to estimate the temperature change of the solution. The time-dependent temperature change is presented in Fig. S17, left. Except for the results in water the changes in temperature obtained from the structural refinement show high uncertainty. This can be explained by the low signal strength of the heat signal compared to the signal caused by structural changes in the solute in MeCN, EtOH, and MeOH. In water the signal caused by solvent heating of 1 K shows a higher amplitude than in the other organic solvents.[5] This is illustrated by Fig. S17, right, which shows the heat signal (solid lines) as refined at 500 ps where we observe the maximal heat response in the time range observed here. The black crosses present the experimental difference scattering for the same time point with subtracted structural signal ($S_{solute} + S_{cage}$).

From the $\Delta S_{\Delta T}$ contribution to the difference signals the temperature increase and the total energy released to solvent can also be estimated.[5] The temperature increase $\Delta T(t)$ for all solvents is presented in in Fig. S17 and from the maximum temperature increase within the observed 500 ps, the energy deposited into solvent heating was determined ($E_{heating}$) and is included in Table S3.

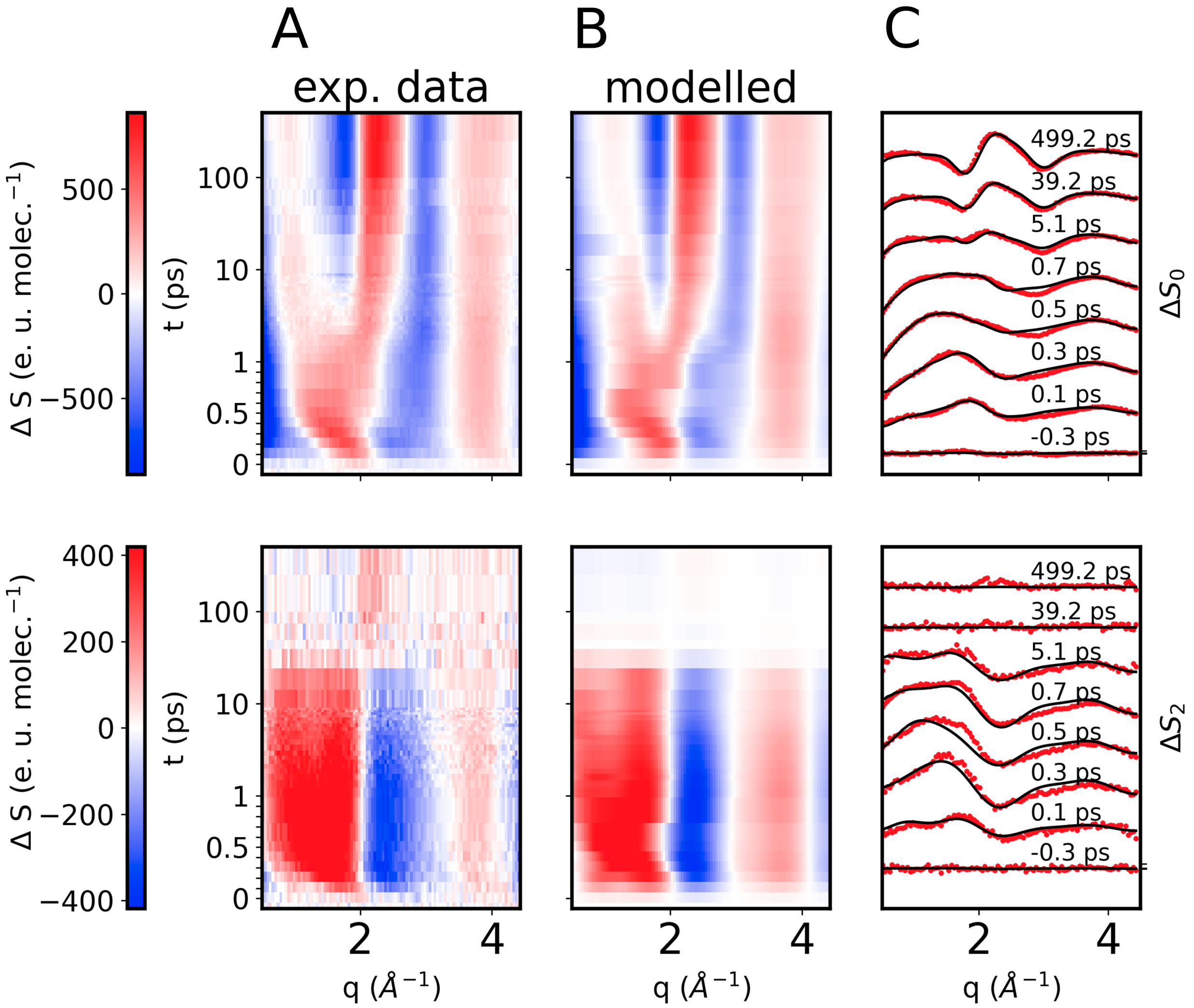

Figure S13: Comparison of modelled and experimental data for I_3^- in water. The top row shows data for ΔS_0, and the bottom row for ΔS_2. Column A: experimental difference scattering. Column B: modelled data. Column C: Both model and experimental data for selected time points.

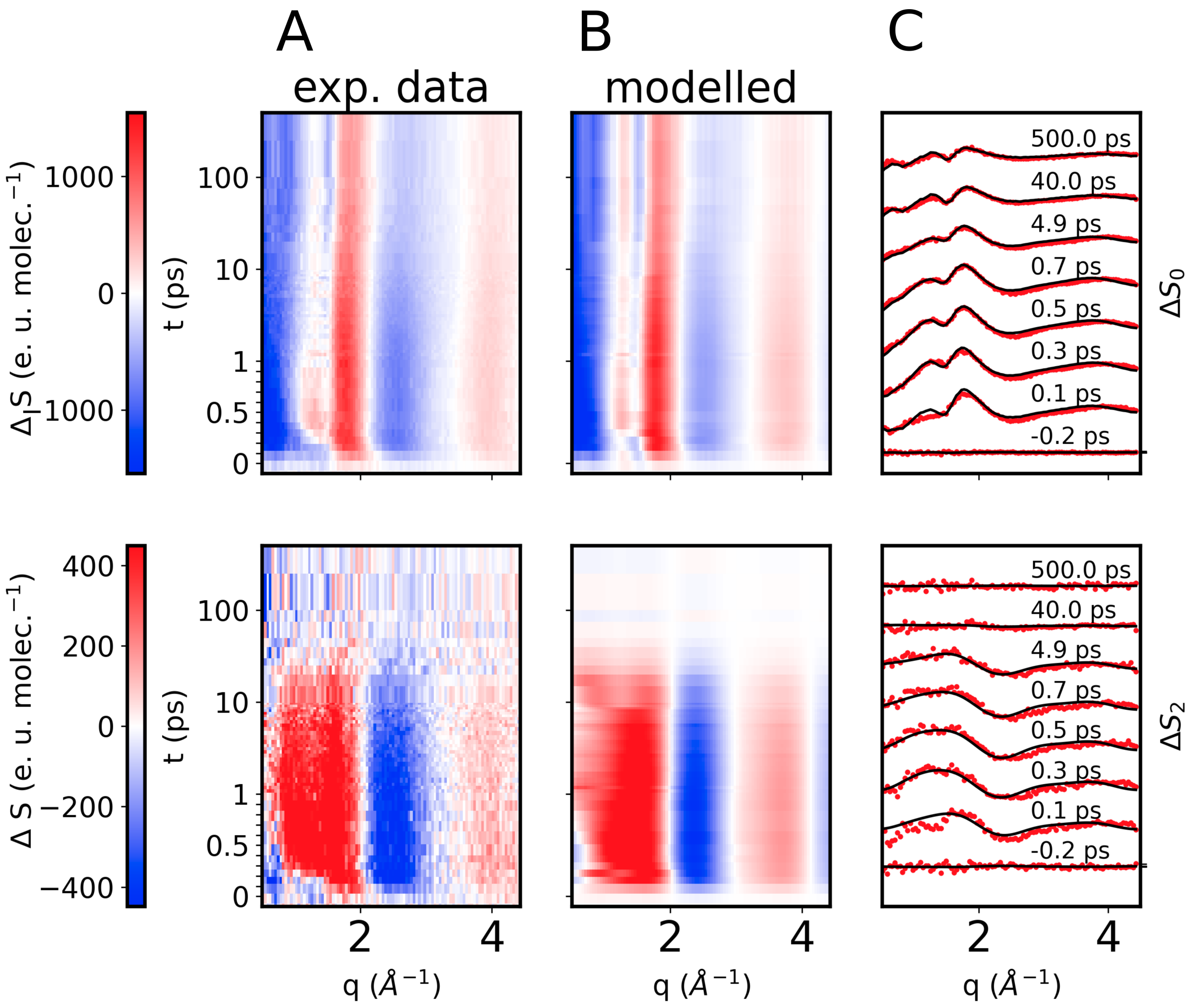

Figure S14: Comparison of modelled and experimental data for I_3^- in ethanol. The top row shows data for ΔS_0, and the bottom row for ΔS_2. Column A: experimental difference scattering. Column B: modelled data. Column C: Both model and experimental data for selected time points.

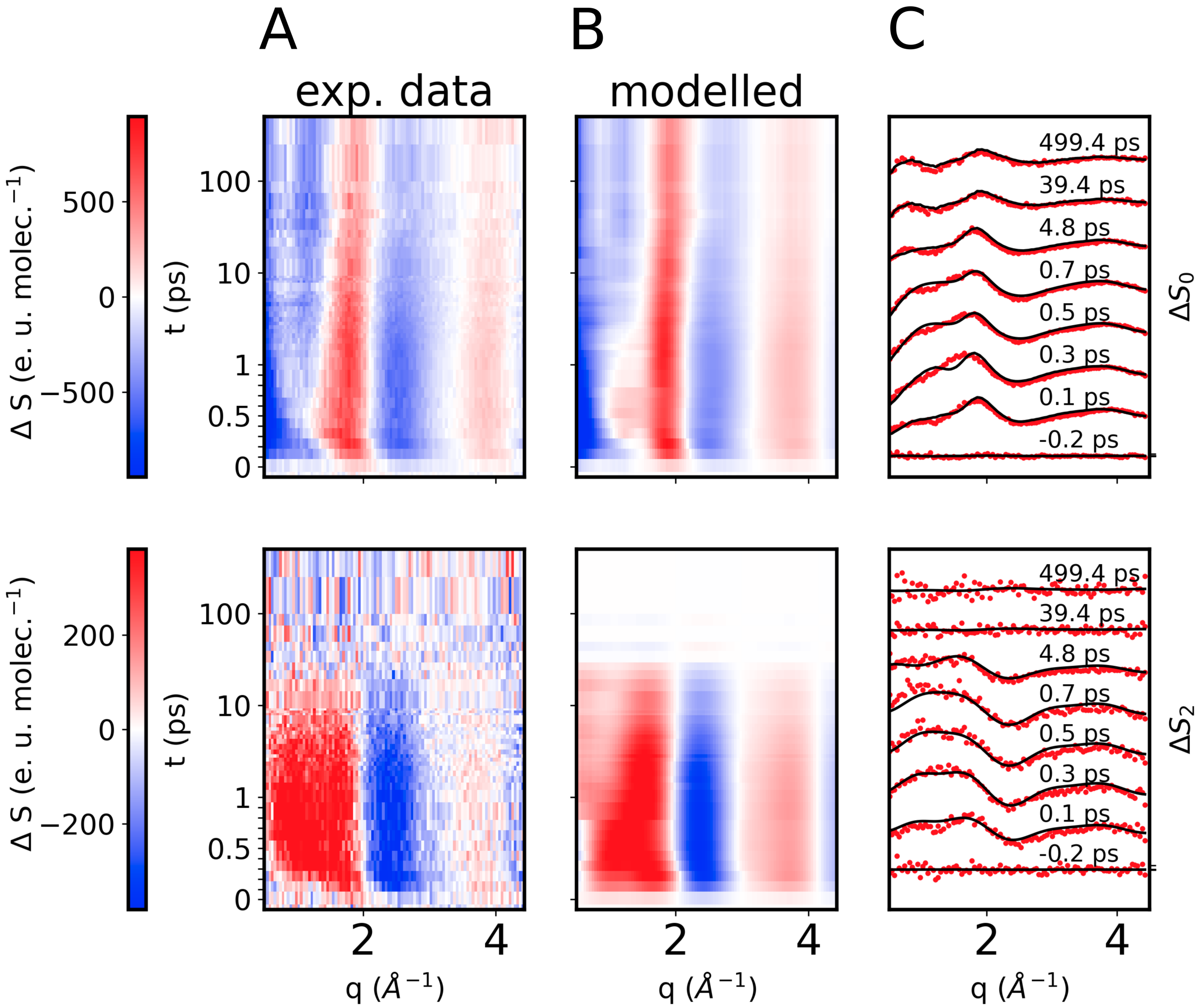

Figure S15: Comparison of modelled and experimental data for I_3^- in methanol. The top row shows data for ΔS_0, and the bottom row for ΔS_2. Column A: experimental difference scattering. Column B: modelled data. Column C: Both model and experimental data for selected time points.

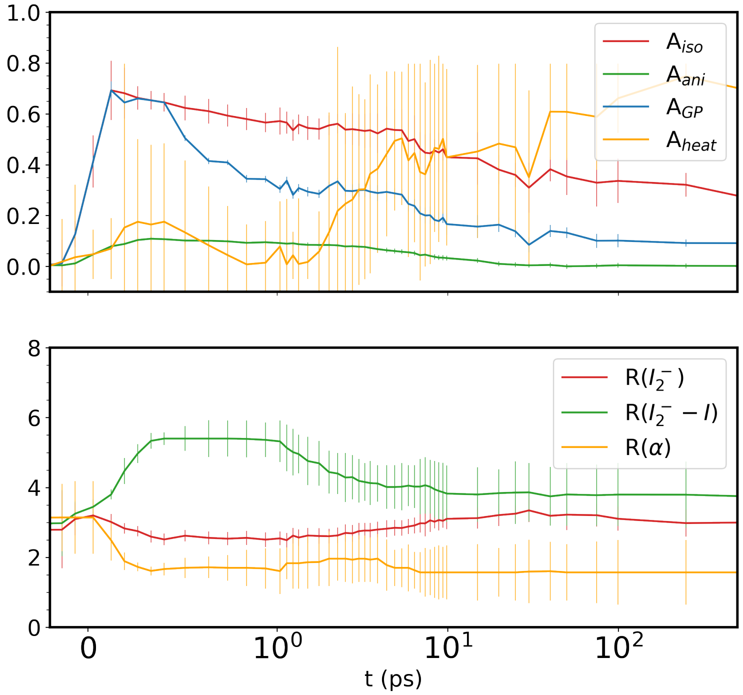

Figure S16: Overview of all paramters refined for the difference scattering data for triiodide in acetonitrile. The top panel shows the amplitudes for the isotropic and anisotropic signal as well as for the fraction of the geminate pair and the amplitude of the heating signal. The lower panel shows the three parameters describing the $I_3{}^-$ structure.

The energy released into solvent heating (600 kJ mol^{-1} to 1100 kJ mol^{-1}) is much higher than the energy of an incident 400 nm photon (299 kJ mol^{-1}) for all solvents. One explanation for the large ΔT observed might be two-photon absorption of iodide in solution, abundant in all solutions (see Supporting InformationTab. S1). This process would lead to ionisation of the iodidide and formation of a solvated electron. The structural changes linked to this process ($I^- \longrightarrow I + e^-$) are linked to the solvent cage, causing only a weak change in scattering signal compared to signals caused by structural changes in $I_3{}^-$.[24] Therefore, this process can be neglected in our analysis.

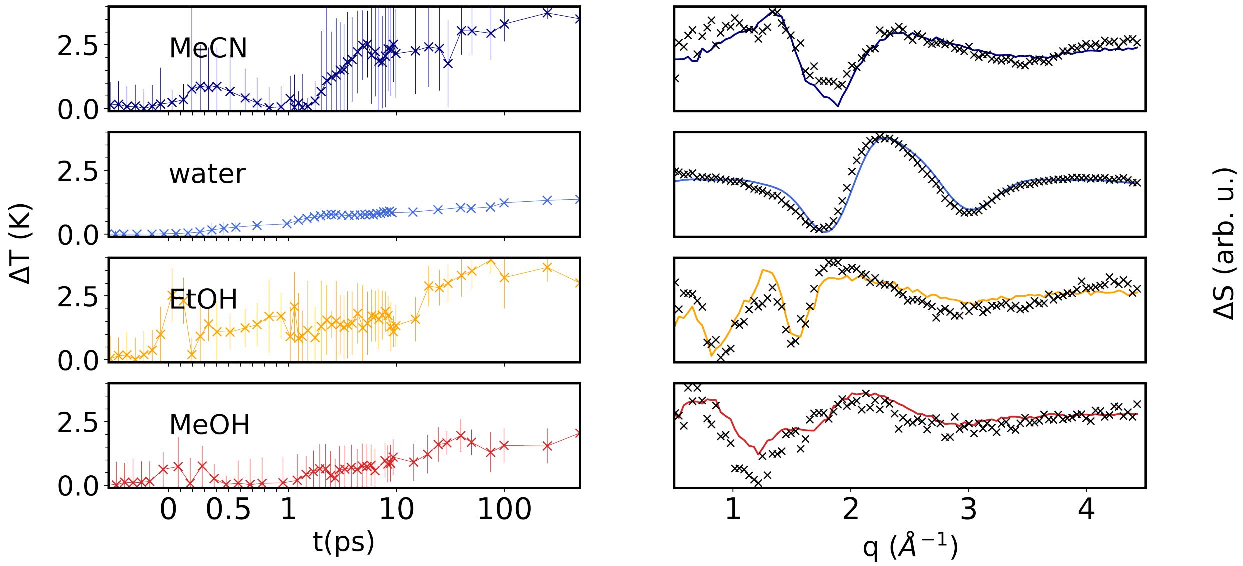

Figure S17: Left: Time dependent change in temperature for the different solvents. Right: Structural signal from the bulk solvent. The black crosses present experimental data ($\Delta S(q)$) at 500 ps from which the refined solute structural signal (as optimised in the refinement). The solid lines show the heating signal multiplied with the amplitude optimised for this time point (solid lines).

Table S3: Temperature increase for all solvents and the corresponding energy release.

	ΔT (K)<br>(K)	$E_{heating}$<br>(kJ mol^{-1})
acetonitrile	3	920
water	1.25	1090
ethanol	3	1010
methanol	2.4	990

Anisotropic population dynamics

Fig. S18 shows the population dynamics obtained from the refinement of isotropic (left) and anisotropic data (right). The excitation fraction observed for the anisotropic signal is much lower than the excitation fraction for isotropic signal due to only a fraction of the excited molecules being aligned. The decay of the anisotropic signal within tens of picoseconds is due to the rotational dephasing of the initially aligned molecules.

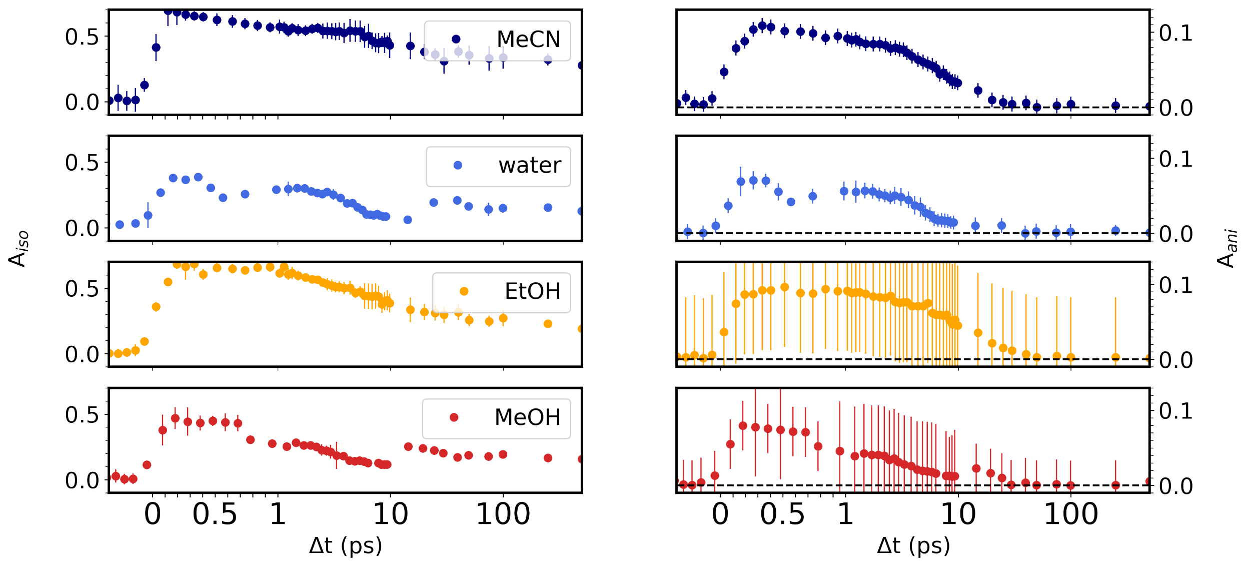

Figure S18: Left: Overall amplitude of the isotropic difference scattering signal, right: overall amplitude of the anisotropic difference scattering signal from the structural refinement.

Kinetics of the GP population

The amplitudes of the GP species obtained from the structural refinement were used to estimate the lifetimes with which the population of this state decays. The decay was fit with an biexponential decay, convoluted with a Gaussian function to model the inital increase. The results are presented in the main paper Fig. 5.

$$f(t) = A_1/2\exp(\frac{-1}{\tau_1}((t-t_0)-\sigma^2/2)) * (1+\frac{\mathrm{erf}(((t-t0)-\sigma^2/\tau)}{(\sqrt{2}\sigma))}) \quad \text{(S12)}$$

$$+A_2/2\exp(\frac{-1}{\tau_2}((t-t_0)-\sigma^2/2)) * (1+\frac{\mathrm{erf}(((t-t_0)-\sigma^2}{\tau_2)/(\sqrt{2}\sigma))}) \quad \text{(S13)}$$

Energy partitioning

From the speed of fragment dissociation (v_{diss}), rotation of the I_2^- fragment (ω), we can estimate the energy partitioning into translational, E_{trans}, and rotational energy, E_{rot}, These are calculated as follows:

$$E_{trans}(\mathrm{kJ\,mol^{-1}}) = 1/2\mu v_{diss}^2 N_A \quad \text{(S14)}$$

With the reduced mass $\mu = \frac{m_1 m_2}{m_1+m_2}$ and Avogadro's number N_A.

The rotational temperature was estimated as:

$$\Theta = I/((\tau_c)^2 3k_B) \quad \text{(S15)}$$

With the rotational correlation time τ_c (the time it takes for α to change by 1 rad, the changes in α are presented in Fig. S21, left), and the moment of intertia $I = mr^2$. For high rotational temperatures (as is the case here), the mean rotational energy of the I_2^- fragment can be calculated as $E_{rot} = \Theta R$.[25] For the calculation of the rotational energy, the average interatomic distance $R(I_2^-)$ before $t = -0.1$ ps was used. The time-dependent changes of $R(I_2^-)$ are presented in Fig. S21, right.

From the heat released into the solvent (ΔT, see previous section), we can further determine the amount of energy released into the solvent, E_{heat}:

$$E_{heat}(\mathrm{kJ\,mol^{-1}}) = \Delta T C_{solv}(\mathrm{J\,K^{-1}\,mol^{-1}})\frac{N_{solv.}}{N_{I_3^-}}/A_{exc} \quad \text{(S16)}$$

With the solvent specific heat capacity (C) and the number of solvent molecules per I_3^- molecule $\frac{N_{solv.}}{N_{I_3^-}}$. The results for all solvents are summarised in Tab. 2, main text.

Error estimation

Due to the regularised refinement, the uncertainty of the optimised parameters cannot be directly determined using the Hessian output from the fminunc function. Instead, the errors were estimated as follows. The χ^2 landscape was determined in the 7D parameter space. A range of 9 values around the optimised parameter from structural refinement were chosen for each parameter. Then, $\chi^2 + f$ was calculated for each possible combination of parameters, leading to 823,543 $\chi^2 + f$ calculations per time point.

For a parameter a, the minimal $\chi^2 + f$, $(\chi^2 + f)_{min}$, for all possible values of a was found. Fig. S19 shows a plot of $(\chi^2 + f)_{min}$ against the values of a. The 1σ uncertainty can be estimated by finding the change in a to achieve a change in $\chi^2 + f$ of 1.[26] The error bars in the figures in the main text and SI show the average of positive and negative uncertainty.

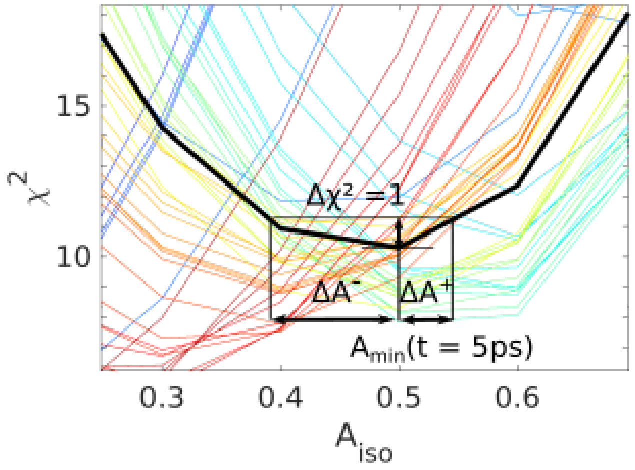

Figure S19: χ^2 curves for the overall scaling parameter from the refinement of the MeCN data. The χ^2 values were determined for a range of values around the optimised solution for all time points (from negative delays (blue) to late delays (dark red). The determination of uncertainty is exemplified for t =500 ps. The 1σ level corresponds to an increase of 1 of the χ^2 which is indicated by the dashed lines. Positive and negative uncertainty (ΔA^- and ΔA^+) are the change of the scaling parameter to cause a change of $\Delta\chi^2 = 1$.

Noise estimation

For calculation of χ^2 as part of the structural refinement, we need to determine the standard deviation σ of the difference scattering signal. σ is expected to show q-dependency as the number of pixels measured depends on the respective q-bin. We used difference scattering from time delays below -0.5 ps where no signal should be recorded to estimate the noise.

$$\sigma(q) = \sqrt{\frac{\sum_{t<-0.2ps}(\Delta S(q,t) - 0)^2}{N_t}} \tag{S17}$$

With the number of time points N_t. Fig. S20 shows the $\sigma(q)$ for anisotropic and isotropic scattering for all solvents.

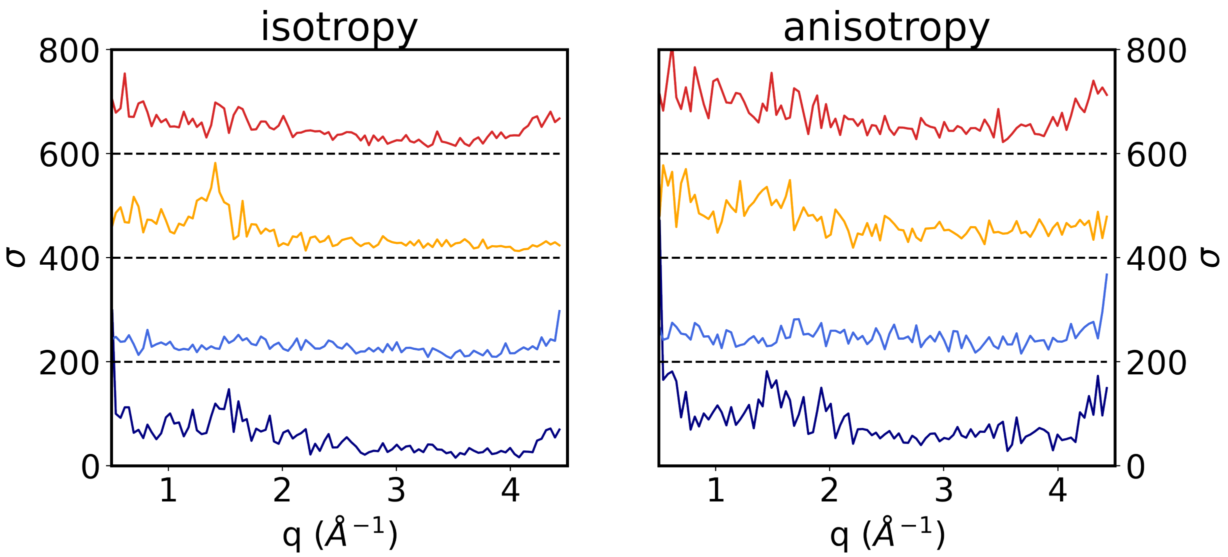

Figure S20: $\sigma(q)$ for all solvents (navy: acetonitrile, light blue: water, orange: ethanol and red methanol). Left: for ΔS_0. Right: for ΔS_2. The curves for the different solvents are offset with 100.

Structural refinement

Fig. S16 shows an overview of all parameters optimised in the structural refinement of the data in acetonitrile with the according error bars. Fig. S21 shows the optimised values for $R(I_2^-)$ and α

for all solvents. The optimised values for $R(I_2^- - I)$ an amplitudes are presented in the main paper (Fig. 5 and Fig. 6) for all solvents.

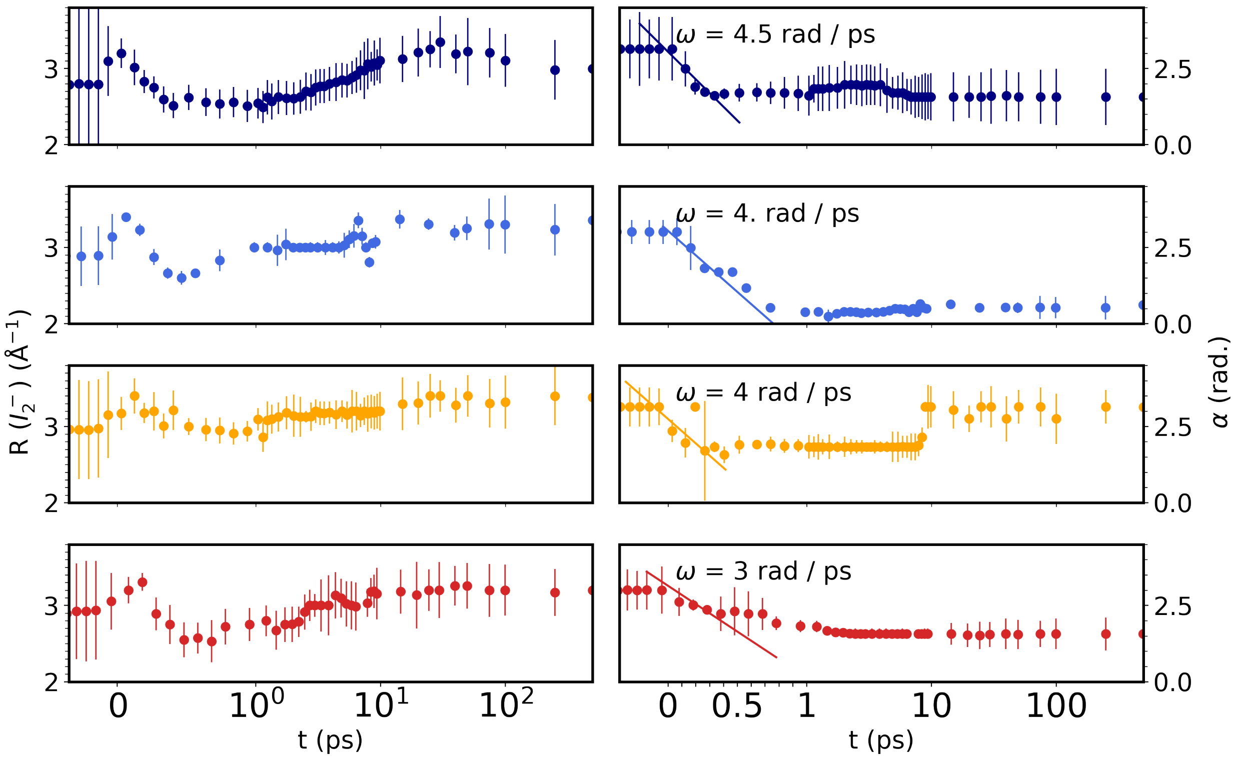

Figure S21: $R(I_2{}^-)$ (left) and α (right as function of delay time determined from the structural refinement.

Three Body Dissociation

In order to exclude possible contributions from three-body dissociation as observed for excitation of $I_3{}^-$ with 266 nm, or in the gas phase, we have simulated difference scattering caused by 3-body dissociation and compared it to simulated difference scattering for two-body dissociation (as assumed in the structural refinement). The results for dissociation in acetonitrile are plotted in Fig. S22. The simulated difference scattering was multiplied with the time-dependent excitation fraction from obtained from the structural refinement, to make the results more comparable. Dissociation was modelled with 5 Å ps^{-1} for the first 700 fs. The experimental data show a much better agreement with the pattern of the simulated two-body dissociation.

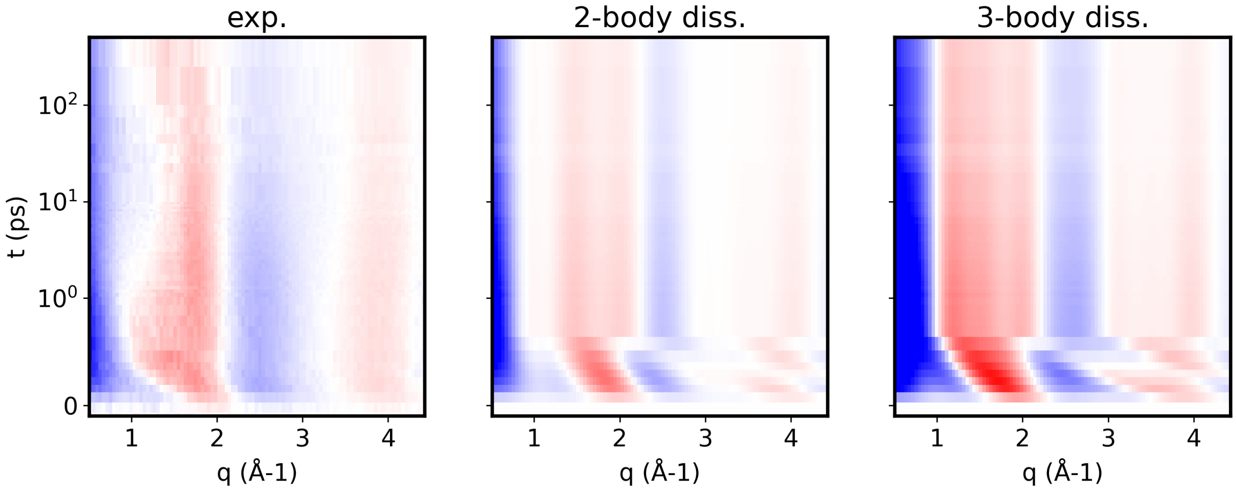

Figure S22: Experimental $\Delta S(q)$ (left) and simulated $\Delta S(q)$ for two-body (middle) and three-body (right) dissociation. Dissociation was modelled with 5 Å ps^{-1} for 700 fs.

Implementation of the Debye-Waller-like factor

As mentioned in the main text we implement a Debye-Waller-like factor (DWF) on the atomic form factors instead of on the molecular form factor as in previous XSS studies.[27,28] Fig. S23 presents a comparison of the results of structural refinement applying both implementations of the DWF. For applying the DWF to the molecular form factor, the solute term was calculated as:

$$S_{solute}(q,\mathbf{R}) = \sum_{i,j} F^*_{I,i}(q) F_{I,j}(q) \operatorname{sinc}(qd_{ij}) \exp[-q^2\sigma^2_{i,j}/3]. \tag{S18}$$

With $\sigma_{i,j}$ being the rmsd of the interatomic distance $d_{i,j}$. For the comparison $\sigma_{i,j}$ was estimated as $\sqrt{\sigma_i}$ with σ_i the rmsd applied in eq. 3 in the main text. Results of the refinement applying the DWF on the atomic form factor (see eq. 3 in the main text) correspond to the results presented in the main text and are plotted as crosses. Results of the refinement applying the DWF to the molecular form factor are plotted as circles, where apart from the implementation of the DWF factor on the molecular form factor, the refinement was implemented as described in the main text. The left panels show the refined interatomic distances, $R(I_2^-)$ in red and $R(I_2^- - I)$ in blue. The right panels show the refined amplitudes for the geminate pair (green) and the non-geminate pair (orange). The results are presented for all solvents and only show minor discrepancies in the refined parameters

using the two implementations of the DWF showing that the main conclusions of this paper do not depend on the implementation of the DWF.

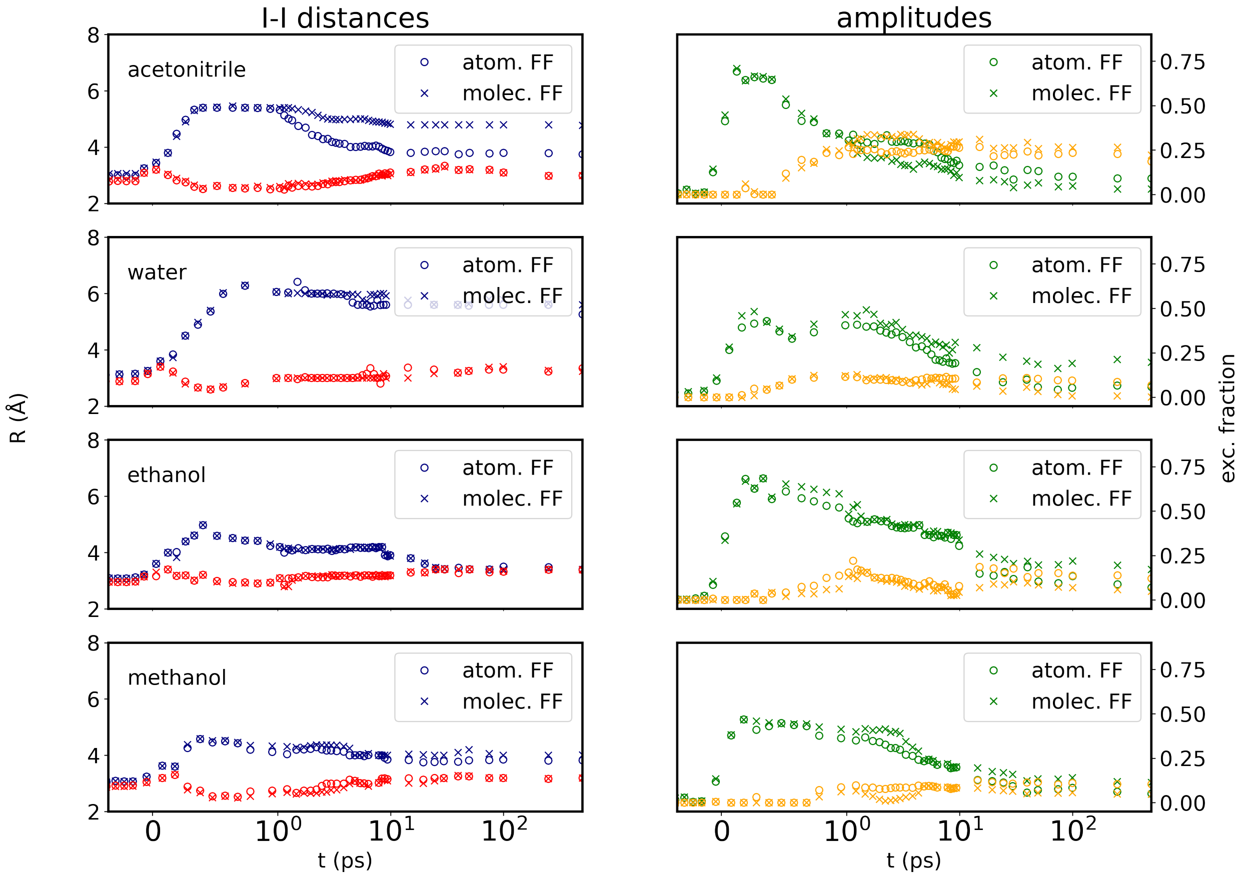

Figure S23: Comparison of the results of structural refinement applying both implementations of the DWF. Results of the refinement applying the DWF on the atomic form factor are plotted as crosses, results of the refinement applying the DWF to the molecular form factor are plotted as circles. The left panels show the refined interatomic distances, $R(I_2^-)$ in red and $R(I_2^- - I)$ in blue. The right panels show the refined amplitudes for the geminate pair (green) and the non-geminate pair (orange).

References

(1) Van Driel, T. B.; Kjær, K. S.; Biasin, E.; Haldrup, K.; Lemke, H. T.; Nielsen, M. M. Disentangling detector data in XFEL studies of temporally resolved solution state chemistry. *Faraday Discussions* **2015**, *177*, 443–465.

(2) Tono, K.; Togashi, T.; Inubushi, Y.; Sato, T.; Katayama, T.; Ogawa, K.; Ohashi, H.; Kimura, H.; Takahashi, S.; Takeshita, K. et al. Beamline, experimental stations and photon beam diagnostics for the hard x-ray free electron laser of SACLA. *New Journal of Physics* **2013**, *15*, 083035.

(3) Kameshima, T.; Ono, S.; Kudo, T.; Ozaki, K.; Kirihara, Y.; Kobayashi, K.; Inubushi, Y.; Yabashi, M.; Horigome, T.; Holland, A. et al. Development of an X-ray pixel detector with multi-port charge-coupled device for X-ray free-electron laser experiments. *Review of Scientific Instruments* **2014**, *85*, 033110.

(4) Haldrup, K.; Christensen, M.; Meedom Nielsen, M. Analysis of time-resolved X-ray scattering data from solution-state systems. *Acta Crystallographica Section A Foundations of Crystallography* **2010**, *66*, 261–269.

(5) Kjær, K. S.; van Driel, T. B.; Kehres, J.; Haldrup, K.; Khakhulin, D.; Bechgaard, K.; Cammarata, M.; Wulff, M.; Sørensen, T. J.; Nielsen, M. M. Introducing a standard method for experimental determination of the solvent response in laser pump, X-ray probe time-resolved wide-angle X-ray scattering experiments on systems in solution. *Phys. Chem. Chem. Phys.* **2013**, *15*, 15003–15016.

(6) Katayama, T.; Owada, S.; Togashi, T.; Ogawa, K.; Karvinen, P.; Vartiainen, I.; Eronen, A.; David, C.; Sato, T.; Nakajima, K. et al. A beam branching method for timing and spectral characterization of hard X-ray free-electron lasers. *Structural Dynamics* **2016**, *3*, 034301.

(7) Wang, J.; Wolf, R. M.; Caldwell, J. W.; Kollman, P. A.; Case, D. A. Development and testing of a general Amber force field. *Journal of Computational Chemistry* **2004**, *25*, 1157–1174.

(8) Benjamin, I.; Banin, U.; Ruhman, S. Ultrafast photodissociation of I3- in ethanol: A molecular dynamics study. *The Journal of Chemical Physics* **1993**, *98*, 8337–8340.

(9) van der Spoel, D.; van Maaren, P. J.; Caleman, C. GROMACS molecule & liquid database. *Bioinformatics* **2012**, *28*, 752–753.

(10) Berendsen, H. J. C.; Grigera, J. R.; Stroatsma, T. P. The Missing Term in Effective Pair Potentials. *J. Chem. Phys.* **1987**, *91*, 6269.

(11) Pronk, S.; Páll, S.; Schulz, R.; Larsson, P.; Bjelkmar, P.; Apostolov, R.; Shirts, M. R.; Smith, J. C.; Kasson, P. M.; Van Der Spoel, D. et al. GROMACS 4.5: A high-throughput and highly parallel open source molecular simulation toolkit. *Bioinformatics* **2013**, *29*, 845–854.

(12) Van Gunsteren, W. F.; Berendsen, H. J. A Leap-Frog Algorithm for Stochastic Dynamics. *Molecular Simulation* **1988**, *1*, 173–185.

(13) Berendsen, H. J.; Postma, J. P.; Van Gunsteren, W. F.; Dinola, A.; Haak, J. R. Molecular dynamics with coupling to an external bath. *The Journal of Chemical Physics* **1984**, *81*, 3684–3690.

(14) Hess, B. P-LINCS: A parallel linear constraint solver for molecular simulation. *Journal of Chemical Theory and Computation* **2008**, *4*, 116–122.

(15) Darden, T.; York, D.; Pedersen, L. Particle mesh Ewald: an N$\cdot$log(N) method for Ewald sums in large systems. *J. Chem. Phys.* **1993**, *98*, 10089–10092.

(16) Essmann, U.; Perera, L.; Berkowitz, M. L.; Darden, T.; Lee, H.; Pedersen, L. G. A smooth particle mesh ewald potential. *J. Chem. Phys.* **1995**, *103*, 8577–8592.

(17) Ashkenazi, G.; Banin, U.; Bartana, A.; Kosloff, R.; Ruhman, S. Quantum Description of the Impulsive Photodissociation Dynamics of I3- in Solution. *Advances in Chemical Physics* **2007**, *100*, 229–315.

(18) Kim, T. K.; Lee, J. H.; Wulff, M.; Kong, Q.; Lhee, H. Spatiotemporal kinetics in solution studied by time-resolved X-ray liquidography (solution scattering). *ChemPhysChem* **2009**, *10*, 1958–1980.

(19) Dohn, A. O.; Biasin, E.; Haldrup, K.; Nielsen, M. M.; Henriksen, N. E.; Møller, K. B. On the calculation of x-ray scattering signals from pairwise radial distribution functions. *Journal of Physics B: Atomic, Molecular and Optical Physics* **2015**, *48*, 244010.

(20) Panman, M. R.; Biasin, E.; Berntsson, O.; Hermann, M.; Niebling, S.; Hughes, A. J.; Kübel, J.; Atkovska, K.; Gustavsson, E.; Nimmrich, A. et al. Observing the Structural Evolution in the Photodissociation of Diiodomethane with Femtosecond Solution X-Ray Scattering. *Physical Review Letters* **2020**, *125*, 226001.

(21) Jena, N. K.; Josefsson, I.; Eriksson, S. K.; Hagfeldt, A.; Siegbahn, H.; Björneholm, O.; Rensmo, H.; Odelius, M. Solvent-dependent structure of the I3- ion derived from photoelectron spectroscopy and Ab initio molecular dynamics simulations. *Chemistry - A European Journal* **2014**, *21*, 4049–4055.

(22) Kim, K. H.; Kim, J.; Lee, J. H.; Ihee, H. Topical Review: Molecular reaction and solvation visualized by time-resolved X-ray solution scattering: Structure, dynamics, and their solvent dependence. *Structural Dynamics* **2014**, *1*, 011301.

(23) Hansen, P. C. The L-Curve and its Use in the Numerical Treatment of Inverse Problems. *in Computational Inverse Problems in Electrocardiology, ed. P. Johnston, Advances in Computational Bioengineering* **2000**, *4*, 119–142.

(24) Vester, P.; Kubicek, K.; Alonso-Mori, R.; Assefa, T.; Biasin, E.; Christensen, M.; Dohn, A. o.; van Driel, T. B.; Galler, A.; Gawelda, W. et al. Tracking structural solvent reorganization and recombination dynamics following e- photoabstraction from aqueous I- with femtosecond x-ray spectroscopy and scattering. *The Journal of Chemical Physics* **2022**, *157*, 224201.

(25) Atkins, P.; de Paula, J. *Physical Chemistry*, 8th ed.; Oxford University Press: Oxford, 2006.

(26) Press, W. H.; Teukolsky, S. A.; Vetterling, W. T.; Flannery, B. P. *Numerical Recipes in C*, 2nd ed.; Cambridge University Press: Cambridge, MA, USA, 1992.

(27) Kim, K. H.; Ki, H.; Oang, K. Y.; Nozawa, S.; Sato, T.; Kim, J.; Kim, T. K.; Kim, J.; Adachi, S. I.; Ihee, H. Global reaction pathways in the photodissociation of I3- ions in solution at 267 and 400 nm studied by picosecond X-ray liquidography. *ChemPhysChem* **2013**, *14*, 3687–3697.

(28) Heo, J.; Kim, J. G.; Choi, E. H.; Ki, H.; Ahn, D.-s.; Kim, J.; Lee, S.; Ihee, H. Determining the charge distribution and the direction of bond cleavage with femtosecond anisotropic x-ray liquidography. *Nature Communications* **2022**, *13*, 522.
